# Structured light analogy of quantum squeezed states

**DOI:** 10.1038/s41377-024-01631-x

**Published:** 2024-10-21

**Authors:** Zhaoyang Wang, Ziyu Zhan, Anton N. Vetlugin, Jun-Yu Ou, Qiang Liu, Yijie Shen, Xing Fu

**Affiliations:** 1 Department of Precision Instrument, Tsinghua University, Beijing, 100084 China; 2 State Key Laboratory of Precision Space-Time Information Sensing Technology, Beijing, 100084 China; 3 Key Laboratory of Photonic Control Technology (Tsinghua University), Ministry of Education, Beijing, 100084 China; 4 Centre for Disruptive Photonic Technologies, School of Physical and Mathematical Sciences & The Photonics Institute, Nanyang Technological University, Singapore, 63737l Singapore; 5 School of Physics and Astronomy, University of Southampton, Southampton, UK; 6 School of Electrical and Electronic Engineering, Nanyang Technological University, Singapore, 639798 Singapore

**Keywords:** Optical physics, Optical techniques

## Abstract

Quantum optics has advanced our understanding of the nature of light and enabled applications far beyond what is possible with classical light. The unique capabilities of quantum light have inspired the migration of some conceptual ideas to the realm of classical optics, focusing on replicating and exploiting non-trivial quantum states of discrete-variable systems. Here, we further develop this paradigm by building the analogy of quantum squeezed states using classical structured light. We have found that the mechanism of squeezing, responsible for beating the standard quantum limit in quantum optics, allows for overcoming the “standard spatial limit” in classical optics: the light beam can be “squeezed” along one of the transverse directions in real space (at the expense of its enlargement along the orthogonal direction), where its width becomes smaller than that of the corresponding fundamental Gaussian mode. We show that classical squeezing enables nearly sub-diffraction and superoscillatory light focusing, which is also accompanied by the nanoscale phase gradient of the size in the order of *λ*/100 (*λ*/1000), demonstrated in the experiment (simulations). Crucially, the squeezing mechanism allows for continuous tuning of both features by varying the squeezing parameter, thus providing distinctive flexibility for optical microscopy and metrology beyond the diffraction limit and suggesting further exploration of classical analogies of quantum effects.

## Introduction

Structured light enables precise control of light’s degrees of freedom and dimensions, being in demand in fundamental physics and optical technologies^1–5^. Due to its multidimensional nature, structured light provides a versatile platform for transferring and testing quantum-inspired concepts with classical light^6–8^. For instance, the vortex beams were used to simulate quantum cat states^9^, Landau levels^10^, and Laughlin matter^11,12^ and to observe the Berezinskii–Kosterlitz–Thouless phase transition, enabling thermodynamics study in photonic light fluid^13^. Moreover, it has been shown that vector beams with spatially inseparable polarization can resemble some properties of the quantum-entangled Bell states^14,15^, suggesting quantum-inspired applications such as local teleportation^16^, turbulence-resilient communication, and encryption^3,17–19^.

**Fig. 1:**
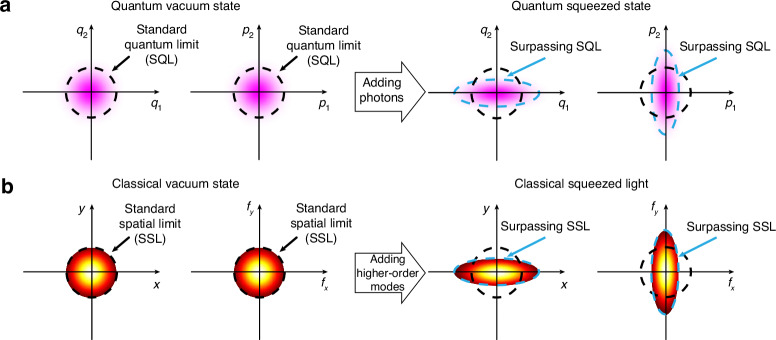
Exploiting the mechanism of quantum squeezing to squeeze classical light. **a** The vacuum state (left diagrams) of a quantum oscillator can be squeezed along one of the directions in the phase space (along ${q}_{2}$ and ${p}_{1}$ directions in right diagrams). Counter-intuitively, the state with more photons—the squeezed state—is less noisy along these directions than the vacuum state with zero photons. The probability distribution in the basis $({p}_{1},{p}_{2})$ is the Fourier transform of the probability distribution in the basis $({q}_{1},{q}_{2})$. **b** For classical structured light, the squeezing methodology can be used to “squeeze” the width of the fundamental Gaussian mode (left diagrams) along one of the directions in the real space $(x,y)$ and the Fourier space $({f}_{x},{f}_{y})$ by adding higher-order modes. The intensity distribution in the basis $({f}_{x},{f}_{y})$ is the Fourier transform of the intensity distribution in the basis $(x,y)$

The search for the classical analogies of quantum phenomena has been primarily focused on the parallelism of structured light and *discrete-variable* (DV) quantum systems. Within this paradigm, a finite Hilbert space of DV systems, characterized by quantum numbers such as energy level and spin, is mimicked by a finite set of spatial modes and polarization states of structured light^20,21^. In contrast, many quantum systems, including optical fields, superconducting circuits, and collective spin of atomic ensembles, are associated with an infinite-dimensional Hilbert space and have canonical variables corresponding to position and momentum (the phase space). The spectrum of these observables is continuous, opposite to the discrete spectrum of DV systems. *Continuous-variable* (CV) systems represent an alternative approach to implementing quantum technologies—from computation and communication to sub-shot-noise metrology^22,23^. The most exploited resource in CV systems is squeezing, where one of the canonical variables (e.g., quadratures of the optical field) is “squeezed,” surpassing the standard quantum limit (the vacuum noise level)—at the expense of the enlarged noise in the orthogonal canonical variable, Fig. 1a. The operation below the standard quantum limit plays a decisive role in high-precision optical measurements, detection of gravitational waves^24–27^, quantum communication^22^, and quantum imaging^28^.

In this work, we develop a classical analogy of the quantum squeezed states. Within this methodology, the infinite-dimensional set of eigenmodes of structured light is used to simulate the Hilbert space of a CV system, while structured light distribution in the transverse plane mimics the behavior of squeezed states in the phase space. Remarkably, the classical analogy of squeezed states exhibits “squeezed” field distribution along one of the directions in real space $(x,y)$ and Fourier space $({f}_{x},{f}_{y})$ compared to the corresponding field distribution of the fundamental Gaussian mode (or “standard spatial limit”), Fig. 1b. Such beam compression along the $x$ and ${f}_{y}$ directions comes at the cost of enlarging the beam size along the ${f}_{x}$ and $y$ directions, similar to quantum squeezing. In this work, we formulate a theoretical framework and develop an experimental apparatus for generating and analyzing such states. We show that classical squeezing holds both in free space propagation and tightly focused conditions where, in the latter case, it is also accompanied by the nanoscale phase gradient (superoscillations)^29–31^ at the deep sub-wavelength level. Therefore, classical squeezed light offers near- and, potentially, sub-diffraction regimes of operation accompanied by sharp phase gradients, both of which can be continuously tuned to meet the requirements of practical super-resolution microscopy, optical metrology, and nanofabrication.

## Results

Fundamentally, quantum-classical analogies arise from similarities in the mathematical description of quantum and classical systems. This occurs when the quantum probability amplitude and the amplitude of classical fields can, under specific conditions, play analogous roles^32^. Here, we exploit a deep connection between Maxwell’s equations in the paraxial approximation and the equations governing the quantized light, where the mathematical isomorphism has been established^33,34^. For instance, the quantum state of two optical modes, which is a primary interest of this work (for a single-mode case, see the Supplementary Material), satisfies the quantum harmonic oscillator equation, $\hat{ {\mathcal H} }|n,m\rangle ={E}_{n,m}|n,m\rangle$^33^, where $\hat{ {\mathcal H} }$ is the Hamiltonian operator, ${E}_{n,m}$ is the discrete eigenvalue (energy) and the state $|n,m\rangle$ corresponds to $n$ photons in mode 1 and $m$ photons in mode 2. Curiously, the paraxial equation can be expressed in the analogous form^34^—the transverse eigenmode equation, ${\hat{ {\mathcal H} }}^{SL}{u}_{n,m}(\xi ,\eta )={C}_{n,m}{u}_{n,m}(\xi ,\eta )$, where ${\hat{ {\mathcal H} }}^{SL}$ is the analogous Hamiltonian operator, $(\xi ,\eta )=(\sqrt{2}x/w(z),\sqrt{2}y/w(z))$ are reduced coordinates, the coefficient ${C}_{n,m}$ is an eigenvalue, and ${u}_{n,m}(\xi ,\eta )$ is a transverse mode function (Supplementary Material). The analytic expression of ${u}_{n,m}(\xi ,\eta )$ in the Cartesian coordinates is the Hermite-Gaussian (HG) mode with the corresponding indices^35^. We note that solutions of quantum and classical equations above constitute infinite-dimensional orthonormal spaces. Consequently, direct mapping can be established: $|n,m\rangle \leftrightarrow {u}_{n,m}(\xi ,\eta )$. Since any two-mode quantum system can be described by a superposition of $|n,m\rangle$ states, this implies the existence of a classical structured light analogy of a diverse family of CV quantum states where ${u}_{n,m}(\xi ,\eta )$ (noted as $|{u}_{n,m}\rangle$ hereafter) modes play a role of $|n,m\rangle$ states.

### Quantum squeezing

Mathematically, the two-mode squeezed vacuum state is generated by applying the squeeze operator to the vacuum state, $|\tau \rangle =\hat{S}(\tau )|0,0\rangle$, where $\hat{S}(\tau )=\exp (-\tau {\hat{a}}_{1}^{\dagger }{\hat{a}}_{2}^{\dagger }+{\tau }^{\ast }{\hat{a}}_{1}{\hat{a}}_{2})$, $\tau$ is the complex number, $|\tau |$ is the squeezing parameter, and $({\hat{a}}_{j}^{\dagger },{\hat{a}}_{j})$ ($j=1,2$) are the ladder operators with the properties ${\hat{a}}_{1}{\hat{a}}_{2}|n,m\rangle =\sqrt{nm}|n-1,m-1\rangle$ and ${\hat{a}}_{1}^{\dagger }{\hat{a}}_{2}^{\dagger }|n,m\rangle =\sqrt{(n+1)(m+1)}|n+1,m+1\rangle$. As mentioned above, the alternative representation of this state can be given in a Fock (number) basis, which reads as $|\tau \rangle =\sum _{n}{c}_{n}|n,n\rangle$ with ${c}_{n}={(-1)}^{n}\exp (in\,{\tanh }^{n}(|\tau |))/\,\cosh (|\tau |)$^33^. A distinctive feature of two-mode squeezed states is that both modes have an equal number of photons as $|\tau \rangle$ has symmetric $|n,n\rangle$-terms only.

### Classical squeezing

The classical ladder operators for structured light, $({\tilde{a}}_{j}^{\dagger },{\tilde{a}}_{j})$, are introduced in a similar fashion^34^: ${\tilde{a}}_{1}{\tilde{a}}_{2}|{u}_{n,m}\rangle =\sqrt{nm}|{u}_{n-1,m-1}\rangle$ and ${\tilde{a}}_{1}^{\dagger }{\tilde{a}}_{2}^{\dagger }|{u}_{n,m}\rangle =\sqrt{(n+1)(m+1)}|{u}_{n+1,m+1}\rangle$. Consequently, the classical analogy of the squeeze operator is $\tilde{S}(\tau )=\exp (-\tau {\tilde{a}}_{1}^{\dagger }{\tilde{a}}_{2}^{\dagger }+{\tau }^{\ast }{\tilde{a}}_{1}{\tilde{a}}_{2})$, with the complex number $\tau$. Therefore, the classical squeezed structured light is generated according to $|{u}_{\tau }\rangle =\tilde{S}(\tau )|{u}_{0,0}\rangle$, where the classical analogy of the quantum vacuum state is the fundamental HG mode $|{u}_{0,0}\rangle$. The classical squeezed state can be represented as an infinite sum of the HG modes $|{u}_{\tau }\rangle =\sum _{n}{c}_{n}|{u}_{n,n}\rangle$, where the amplitudes ${c}_{n}$ are “borrowed” from the quantum squeezed state (see “Methods”). Notably, the classical squeezed structured light inherits remarkable properties of the quantum squeezed state, which we explore theoretically and experimentally in the subsequent sections.

### Beating the standard spatial limit with classical light

**Fig. 2:**
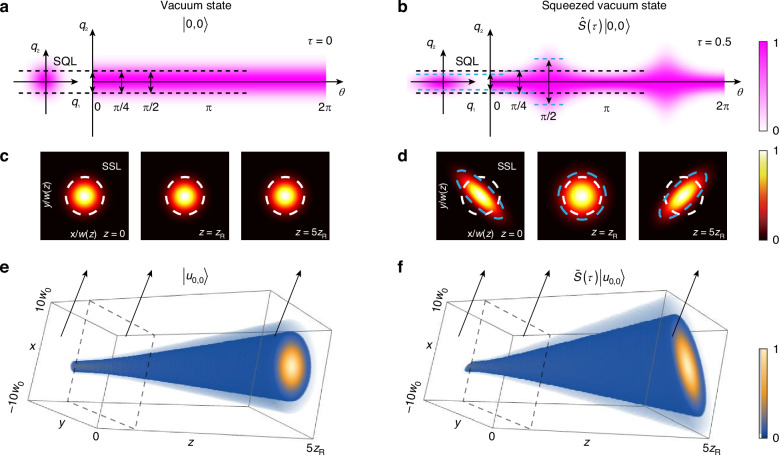
Quantum squeezed state and its structured light analogy. **a**, **b** The quantum probability density distributions for vacuum (**a**) and squeezed vacuum (**b**) states, where the black (blue) dashed lines mark the noise level of the vacuum (squeezed) state. The angle $\theta$ varies from $0$ to $2\pi$; ${q}_{1}$ and ${q}_{2}$ are field quadratures (canonical variables). **c**, **d** The transverse modes of the corresponding structured light analogies (simulation) at different planes perpendicular to $z$, where $(x/w(z),y/w(z))$ varies from $-2$ to 2; white (blue) dashed line marks the waist of Gaussian beam (classical squeezed structured light). **e**, **f** The propagation evolution of the Gaussian and classical squeezed beams; $z$ varies from $0$ to $5{z}_{R}$, $(x,y)$ varies from $-10{w}_{0}$ to $10{w}_{0}$, ${w}_{0}$ is the beam waist at $z=0$, ${z}_{R}$ is the Rayleigh range. SQL standard quantum limit, SSL standard spatial limit

The standard quantum (shot noise) limit (SQL) is inevitable for classical states of light, including single-frequency laser, restricting the precision of optical measurements and imaging. This limit—the ultimate result of quantum vacuum fluctuations—holds for single and multi-component systems. For instance, let us consider two optical modes (e.g., two traveling waves that differ in frequency, polarization, or propagation direction) described by canonical variables, or quadratures, $({q}_{1},{p}_{1})$ and $({q}_{2},{p}_{2})$, accordingly^36^ (see “Methods”). If these modes are in a vacuum state $|0,0\rangle$, the joint measurement of two quadratures, say ${q}_{1}$ and ${q}_{2}$, follows isotropic Gaussian distribution, Fig. 2a. Here, the left subfigure depicts the state’s probability density distribution in the basis $({q}_{1},{q}_{2})$, whereas the right subfigure shows this distribution as a function of the polar angle $\theta$, where $\theta =\arctan ({q}_{1}/{q}_{2})$^37^.

The quantum squeezed vacuum state, $|\tau \rangle$, overcomes the SQL: squeezing suppresses noise in one of the quadratures at the cost of increasing noise in another quadrature, as shown in Fig. 2b for $\tau =0.5$ (left subfigure). The resulting elliptical distribution’s minor axis (blue dashed line) is smaller than the SQL (black dashed line). With varying polar angle $\theta$, the noise oscillates between these two extreme values (Fig. 2b, right subfigure).

As previously mentioned, the vacuum state finds a trivial classical analogy—the fundamental HG mode, $|{u}_{0,0}\rangle$. The mode’s transverse profiles at various planes are shown in Fig. 2c. The profiles are drawn in the reduced coordinate frames $(x/w(z),y/w(z))$, with the beam waist $w(z)$ changing with the longitudinal coordinate $z$. This beam waist (white dashed contours) is regarded as the counterpart of the SQL and, hence, called the “standard spatial limit” (SSL). In contrast, the classical squeezed structured light, $|{u}_{\tau }\rangle$, exhibits an elliptical beam waist, Fig. 2d. Remarkably, the waist is “squeezed” along one of the directions and is smaller than that of the fundamental HG mode. The blue dashed contour highlights that the classical squeezed structured light beats the SSL, shown by the white dashed contour.

The longitudinal evolution of the fundamental HG mode and classical squeezed state is illustrated in Fig. 2e, f, respectively. Light distribution becomes broader during propagation due to the diffraction, necessitating the use of the reduced coordinate frame in Fig. 2c, d. Furthermore, the direction of squeezing oscillates periodically along the propagation direction (Fig. 2f), following the $\theta$-evolution of the quantum squeezed state (Fig. 2b).

### Generation and detection of classical squeezed light

Beyond the mathematical framework, the structured light analogy of squeezed states can benefit from the experimental methods of quantum optics. In this section, we develop the parallelisms between generating and detecting quantum and classical squeezed light.

**Fig. 3:**
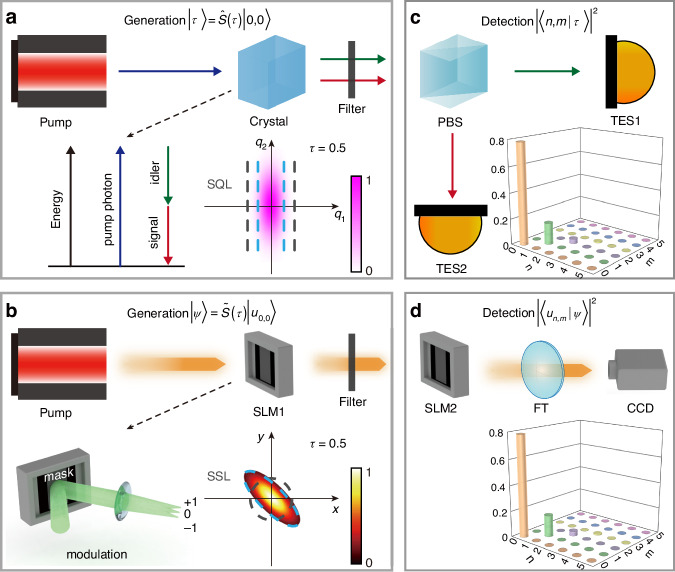
Experimental generation and detection of quantum and classical squeezed light. **a** Generation and **c** detection of quantum squeezed vacuum state. **b** Generation and **d** detection of classical squeezed structured light. PBS polarizing beam splitter, TES transition edge sensor, SLM liquid-crystal spatial light modulator, FT Fourier transform, CCD charge-coupled device camera

The quantum squeezed vacuum state is routinely generated in the spontaneous parametric down-conversion (SPDC) process, taking place in a non-linear crystal exposed to a pump laser. In this process, one laser photon can generate two photons of lower frequency in two distinct (“signal” and “idler”) modes where one photon is always emitted to the signal mode, and the other photon is always emitted to the idler mode. These two modes differ in polarization (as in Fig. 3a), direction, or frequency. If the pump power is sufficiently high, it can stimulate the higher-order processes in which two or more laser photons simultaneously experience such transformation. As a result, the initial vacuum state of the signal and idler modes becomes populated synchronously, generating the squeezed vacuum state $|\tau \rangle =\sum _{n}{c}_{n}|n,n\rangle$ with proper coefficients ${c}_{n}$ (see above). The laser power, non-linear susceptibility of the crystal, interaction length, and phase matching define the squeezing parameter. After the crystal, the pump laser is filtered out.

To generate the classical squeezed structured light, we replace the non-linear crystal in the above scheme with the liquid-crystal spatial light modulator (SLM1), Fig. 3b, loaded with the specially prepared numerical mask (“Methods”). The squeezing takes place in the +1 diffraction order, while all other diffraction orders are filtered out. The mask defines the squeezing parameter, which, importantly, can be continuously varied in a wide range, providing flexibility in the squeezing effect and resulting field patterns.

The quantum squeezing can be experimentally quantified as shown in Fig. 3c. Here, the signal and idler modes are separated using a polarizing beam splitter (PBS), and each output port of the PBS is monitored by a photon-number resolving detector, such as the transition edge sensor. In the photon-number correlation measurements, the coefficients ${|{c}_{n,m}|}^{2}={|\langle n,m|\tau \rangle |}^{2}$ are recovered^38^ (“Methods”). The zero off-diagonal terms are a distinctive feature of the squeezed vacuum state, ${|{c}_{n,m}|}^{2}=0$ for $n\,\ne\, m$.

The classical squeezing can be measured in a similar fashion by replacing the PBS with the second liquid-crystal spatial light modulator (SLM2) and replacing the photon-number resolving detectors with the charge-couple device (CCD) camera, as shown in Fig. 3d. The SLM2 was loaded with the masks of conjugation of different HG modes, while CCD records the modal spectrum ${|\langle {u}_{n,m}|{u}_{\tau }\rangle |}^{2}$ (“Methods”). The classical modal spectrum reproduces the quantum correlations for the same squeezing parameter ($\tau =0.5$ in Fig. 3).

### Classical squeezed light in real space

The quantum squeezed vacuum state has a remarkable ability to surpass the SQL. The classical squeezed light inherits this ability by overcoming the SSL, as experimentally demonstrated in this section.

The classical squeezed structured light is generated by diffracting the 488 nm laser (continuous-wave) on the programmed hologram (“Methods”) as described in the previous section (Fig. 3b). After the reflection from SLM1, the beam is focused by a weakly focusing lens (focal length of 200 mm). We set the value of the squeezing parameter to 0.78 and only retained the eleven HG modes in lowest orders in the state decomposition of $|{u}_{\tau }\rangle$, since negligible coefficients accompany the higher-order modes.

**Fig. 4:**
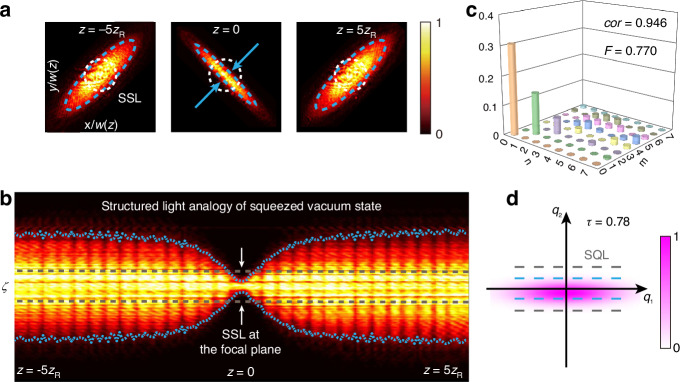
Classical squeezed light in real space. **a** The transverse modes of classical squeezed structured light at $z=-5{z}_{R}$, $0$, and $5{z}_{R}$, respectively. The blue arrows mark the squeezing direction at $z=0$. White and blue dashed lines are the same as in Fig. 2d. **b** The intensity distributions at $\zeta -z$ plane, where $z$ ranges from $-5{z}_{R}$ to $5{z}_{R}$, $\zeta$ is the normalized radial position along the squeezing direction at $z=0$ (the anti-diagonal direction at $z=0$). **c** The detected modal spectrum of the classical squeezed light. **d** The probability density distributions of the corresponding quantum squeezed state (simulation), with $\tau =0.78$

In the first set of measurements, we remove the SLM2 and directly image the transverse profile of the beam using the CCD camera (pixel size of 6.5 μm) at different planes along the propagation direction. In agreement with the simulations in Fig. 2f, the beam’s profile oscillates between diagonal elliptical, circular, and anti-diagonal elliptical shapes; a few of those profiles are shown in Fig. 4a. The different phase dependencies of the HG modes explain this evolution of the squeezing ellipse. At the same time, the beam’s width changes with the propagation distance, and the maximum squeezing occurs at the focal plane, $z=0$.

From the beam’s profile measurements at different planes, we evaluate the normalized radial size $\zeta =r(z)/w(z)$. Here, $r(z)$ is the size of the beam profile along the squeezing direction (the direction is defined as the orientation of the minor axis of the elliptical profile at $z=0$), and $w(z)$ is the beam waist of the fundamental HG mode. The normalized radial size $\zeta$ as the function of propagation direction $z$ is shown in Fig. 4b. Qualitatively, the dynamics of $\zeta$ reproduce the dynamics of quantum squeezing as a function of $\theta$ (Fig. 2b). Importantly, the classical squeezed structured light surpasses the smallest SSL (defined at $z=0$) in the interval $(-0.3{z}_{R},0.3{z}_{R})$, beating the weakly focused fundamental HG mode in this interval.

In the second set of measurements, now with the SLM2 in place as shown in Fig. 3d, we measure the modal spectrum of the classical squeezed structured light, following the procedure described in the previous section. The resulting distribution is shown in Fig. 4c. This measurement confirms the dominant contribution of the first three modes: $|{u}_{0,0}\rangle$, $|{u}_{1,1}\rangle$, and $|{u}_{2,2}\rangle$. Notably, the distribution is diagonal, which agrees with the number state representation of squeezed states. Based on this distribution, the correlation coefficient $cor$ between the theoretical and experimental distributions is estimated to be 0.946, while the fidelity is estimated to be 0.770 (“Methods”). We attribute the appearance of small non-diagonal terms and the deviation of $cor$ and $F$ from unity to the imaging system’s aberration and the CCD camera’s noise. For the context, we also plot the probability density distribution for the quantum squeezed states with the same level of squeezing in Fig. 4d, where the gray (blue) dashed line marks the SQL (the squeezed noise).

### Classical squeezed light in Fourier space

In the previous section, we demonstrated that classical squeezed light beats the SSL in real space. At the same time, in the Fourier space (being focused by a microscope objective), such light can operate at near- and sub-diffraction limit, as we discuss in this section.

In the tight focusing experiment, we send the classical squeezed light (after the SLM1) through a lens and the microscope objective, composing a 4f system with 60× magnification (“Methods”). In this configuration, the effective pixel size is 108 nm (=6.5 μm/60). The numerical aperture (NA) of the microscope objective is 0.95. Justified by the continuous and smooth variation of the intensity distribution recorded in the experiment, we exploit an interpolation function to restore the intensity distribution at the subpixel level of 23 nm from the image recorded at the effective pixels of 108 nm. The schematic diagram of the tightly focusing experiment and the phase retrieval standard method are presented in “Methods” and Supplementary Material, respectively.

**Fig. 5:**
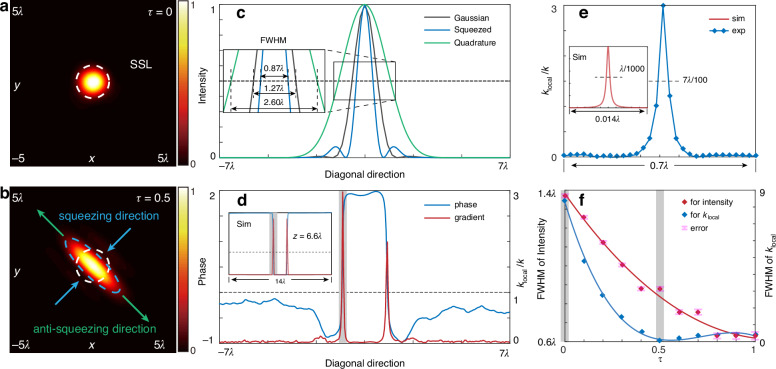
Tunable deep-sub-wavelength features of classical squeezed structured light in tightly focused conditions. **a**, **b** The tightly focused intensity patterns with $\tau =0$ (the fundamental HG mode) and $\tau =0.5$ (classical squeezed structured light), respectively, where $(x,y)$ ranges from −5λ to 5λ. **c** The intensity along the diagonal and anti-diagonal directions, where the gray curve corresponds to $\tau =0$, while the blue and green curves correspond to $\tau =0.5$. **d** The phase (blue line) and local wavenumber (red line) of structured light along the squeezing direction retrieved from the experiment. Inset: simulated phase gradient at *z* = 6.6λ plane. The gray regions highlight superoscillatory structures with the local wavenumber larger than the free-space wavenumber $k$. **e** The local wavenumber in the gray region in (**d**) retrieved from the experiment (blue line) and simulations (red line in the inset). **f** Tuning the beam spot size and the local wavenumber with the squeezing parameter $\tau$ (simulations). Here, the FWHM of ${k}_{local}$ is measured in λ/100

The tightly focused patterns of structured light with $\tau =0$ (fundamental mode) and $\tau =0.5$ (squeezed) are shown in Fig. 5a, b, respectively. Importantly, the tight focusing process preserves the squeezing—the beam waist of the classical squeezed light (blue dashed line) is noticeably smaller than that of the fundamental HG mode (white dashed line). The analysis of the diagonal and anti-diagonal sections of these intensity maps, Fig. 5c, reveals that the full width at half maximum (FWHM) in the squeezing direction (0.87*λ*) is one and a half times smaller than that of the Gaussian beam (1.27*λ*). Squeezing comes at the cost of doubling the spot size in the anti-diagonal direction (FWHM 2.60*λ*). We note that simulations based on the parameters of our experiment (but free of aberrations) predict the FWHM in a squeezing direction at the level of 0.6*λ*, approaching the diffraction limit. It is important to note the difference between the diffraction limit and the standard spatial limit. The diffraction limit describes the resolution limit of imaging systems, defined as *λ*/(2NA)^39^, where *λ* is the wavelength, and NA is the numerical aperture. The standard spatial limit is defined as the beam waist of the Gaussian beam—the structured light counterpart of the standard quantum limit. Therefore, classical squeezed structured light can surpass the standard spatial limit if the squeezing parameter is greater than zero. However, to overcome the diffraction limit, significantly large squeezing parameters are necessary. Experimentally, this would involve incorporating higher-order eigenmodes, which is beyond the scope of this paper.

### Superoscillatory structure of classical squeezed light

Curiously, despite beam squeezing, the tightly focused classical squeezed light exhibits large local wavenumbers, exceeding the free-space wavenumber $2\pi /\lambda$, and, accordingly, deep sub-wavelength superoscillatory structure^40^. We note that these properties go beyond the quantum-classical analogy. The local wavenumber *k*_local_ is defined as the gradient of the phase *P*(*x*, *y*), *k*_local_ = |∇*P*(*x,y*)|. Both phase and local wavenumber are shown in Fig. 5d as a function of position along the diagonal direction. Here, the dimension of the local wavenumber is 1/*k* and the superoscillatory regions appear for *k*_local_/*k* > 1 (the corresponding area is highlighted with a gray color). Zooming in this region, Fig. 5e shows that the FWHM of the local wavenumber is 7*λ*/100, which is about 34 nm (in simulation, the FWHM of the local wavenumber is *λ*/1000 for the parameters of our experiment).

Importantly, the FWHM of both intensity and local wavenumber can be continuously tuned by changing the squeezing parameter *τ*. The results of simulations shown in Fig. 5f confirm that the field features become smaller for larger *τ* (*k*_local_/*k* also exhibits weak oscillation behavior). Note that, the simulations accounting for the parameters of experimental setup are limited within the range of *τ* ≤ 0.5. For *τ* > 0.5, the size of the tightly focused spot approaches the size of the effective CCD pixel, thus increasing the measurement error (pink labels in Fig. 5f), which becomes large compared to the signal.

## Discussion

In this work, we have introduced the framework for characterizing and experimentally realizing classical analogies of quantum squeezed states, demonstrating that quantum and classical squeezed states exhibit similar behavior in phase space (quantum) and real space (classical), respectively. We have exploited this parallelism to establish the squeezing of the structured light beam along one of the directions in the transverse plane to overcome the standard spatial limit, which serves as an analogy of the standard quantum limit.

These results suggest that structured light is a robust and easily accessible platform for studying and demonstrating the properties of quantum states of CV quantum systems. Clearly, the developed analogy can be extended to a broader range of quantum states. To illustrate this, we provide an analysis of the classical analogies of quantum squeezed number state, displaced squeezed vacuum state, and displaced squeezed number state in the Supplementary Material. Notably, substantial complications are required in the setup (Fig. 3a, c) to generate and detect such complex quantum states, and, to the best of our knowledge, none of these states has been demonstrated experimentally. Intriguingly, the generation and detection of the classical counterparts can be implemented by simply changing the numerical masks (“Methods”). Thus, a classical setup (Fig. 3b) can serve as a testbed for demonstrating and exploring complex quantum experiments. Besides, the classical analogies of a single-mode squeezed state^41^ can be studied similarly using classical structured light, as detailed in Supplementary Material. Moreover, the developed framework enables the exploration of structured light analogies of other families of states, such as the cat state^9^, GHZ state^42^, and NOON state, paving the way for further exploration of multidimensional structured light, quantum-classical connection, and its advanced applications.

Importantly, our experiments and simulations have established that the classical squeezing is preserved after the tight focusing. As a result, the spot size of classical squeezed structured light can reach near the diffraction limit, while the local wavenumber can surpass the diffraction limit. The spot size of classical squeezed structured light beats the corresponding SSL, while we demonstrated experimentally (0.87*λ*) and in simulations (0.6*λ*) operation near the diffraction limit. Surpassing the diffraction limit requires large squeezing parameters and correspondingly higher-order eigenmodes, which, in principle, could be achieved in a more advanced setup. Moreover, we have found that tightly focused classical squeezed light exhibits a local phase gradient (superoscillation) at a deep sub-wavelength level (*λ*/100 in the experiment and *λ*/1000 in simulations). Although a local phase gradient at the level of *λ*/100 can be realized in different ways^43^, having *both* a near or sub-diffraction beam spot and a sharp phase gradient is not trivial and can provide additional advantages for optical measurements. For instance, while superimposing two opposite-handed vortex beams allows the generation of a sharp local phase gradient, the resulting spot size will be much larger than in our experiment. Specifically, the spot size of superimposing two opposite-handed vortex beams is about $\sqrt{p+2l+1}{w}_{0}$, where *w*_0_ is the beam waist of the Gaussian beam^44^, and *p* and *l* are the indices of the vortex. Accordingly, the spot size cannot be smaller than *w*_0_, and it follows square root scaling with increasing vortex indices. In contrast, the spot size of squeezed structured light scales as *w*_0_ exp(−|*τ*|) along the squeezing direction. The realizable squeezing parameters |*τ*| depend on the experimental setup, but even moderate values of |*τ*| allow a spot size significantly smaller than *w*_0_ (the SSL).

The structured light analogy of squeezed states offers a robust method for operation at the near- and sub-diffraction limit and generating superoscillatory structures, providing powerful tools for ultra-precise measurement^40^, microscopy^45^, super-resolution imaging^46^, and nanofabrication^47^. In the context of imaging, for instance, classically squeezed light can replace or complement the method of imaging of 1D objects by a set of masks^48^ where the resolution in one direction is enhanced by sacrificing resolution in the orthogonal direction. Moreover, the classical squeezed light provides a unique mechanism for *continuous tuning* of spot and phase gradient sizes via adjusting the squeezing parameter. Indeed, in general, one does not require as small features (spot and phase gradient) as possible but rather find a compromise between the size of those features and the corresponding disadvantages. In the case of metrology with the sharp phase gradient^29^, this compromise includes the energy consideration: the sharper the phase gradient, the smaller the energy it carries, and, accordingly, the longer the integration time to extract the information about the parameter of interest. By adjusting the size of the phase gradient in situ, one could reduce the integration time, thus enabling the metrology of dynamic systems while keeping the phase gradient sharp enough for a particular measurement scenario. Similarly, the tunable beam spot size is desirable in manufacturing applications (e.g., lithography), where the fabrication of large components can be assembled with the larger beam spot, hence faster, while for components with smaller features, the beam can be squeezed accordingly.

In this work, we have demonstrated the classical squeezed light in free space. Yet, this concept could benefit from implementation in integrated platforms, including optical fibers and waveguides, parity-time (PT), and anti-PT symmetric Hamiltonian system^49,50^. Since the diffraction is naturally surpassed in such systems, the quantum-to-classical correspondence can be established between *θ*-evolution (quantum) and *z*-propagation (classical), with possible implications for optical sensing and computing applications^51,52^.

Also, one of the possible extensions of this work could be exploring the classical analogies of quantum states on the basis of modes other than HG (e.g., Laguerre-Gaussian, Ince-Gaussian, and Bessel-Gaussian modes). Moreover, the astigmatic Gaussian beams focused by a single cylindrical lens could also exhibit classical squeezing (Supplementary Material), suggesting to investigate further the connection between quantum states and classical astigmatic optics.

Beyond the interest in classical optics, the complex spatial modes of squeezed structured light hold notable advantages for applications in quantum optics^8^. For instance, the interference of structured photons shaped into the squeezed spatial profiles on a beam splitter^53^ or in free space^54^ would allow for generating entangled, highly correlated states of multidimensional structured light. Indeed, such a basis exhibits multiple degrees of freedom, including squeezing parameter *τ*, displacement parameter *α*, and mode number *N*, which could incorporate the methods of quantum CV states into the teleportation and communication using structured light^55^.

## Materials and methods

### The probability distribution of quantum squeezed states

The annihilation ${\hat{a}}_{j}$ and creation ${\hat{a}}_{j}^{\dagger }$ operators are defined via the canonical variable operators ${\hat{p}}_{j}$ and ${\hat{q}}_{j}$ (${\hat{q}}_{j}$ and ${\hat{p}}_{j}$ correspond to observables *q*_*j*_ and *p*_*j*_) as^33^:

1 $${\hat{a}}_{j}=\frac{{\hat{q}}_{j}+i{\hat{p}}_{j}}{\sqrt{2}}$$

2 $${\hat{a}}_{j}^{\dagger }=\frac{{\hat{q}}_{j}-i{\hat{p}}_{j}}{\sqrt{2}}$$

The structured light counterparts of the ladder operators $({\hat{a}}_{j}^{\dagger },{\hat{a}}_{j})$ are defined as^34^:

3 $${\tilde{a}}_{j}=\frac{{\tilde{q}}_{j}+i{\tilde{p}}_{j}}{\sqrt{2}}$$

4 $${\tilde{a}}_{j}^{\dagger }=\frac{{\tilde{q}}_{j}-i{\tilde{p}}_{j}}{\sqrt{2}}$$

where ${\tilde{p}}_{j}=-i\frac{\partial }{\partial j}$ and ${\tilde{q}}_{j}=j$, where *j* = *ξ*, *η* represents two orthogonal directions in a two-dimensional space.

The wavefunction of two-mode vacuum states in the basis (*q*_1_, *q*_2_) can be expressed as^36^:

5 $${\varPsi }_{vac}({q}_{1},{q}_{2})=\frac{1}{\sqrt{\pi }}\exp \left(-\frac{{q}_{1}^{2}}{2}-\frac{{q}_{2}^{2}}{2}\right)$$

The probability distribution ${|{\varPsi }_{vac}({q}_{1},{q}_{2})|}^{2}$ is plotted in the left subfigure of Fig. 2a. The wavefunction of two-mode vacuum states in the momentum basis, ${\varPsi }_{vac}({p}_{1},{p}_{2})$, is the Fourier transform of ${\varPsi }_{vac}({q}_{1},{q}_{2})$^36^.

The wavefunction of two-mode squeezed vacuum states in the basis $({q}_{1},{q}_{2})$ could be expressed as^36^:

6 $${\varPsi }_{sq}({q}_{1},{q}_{2})=\frac{1}{\sqrt{\pi }}\exp \left[-\frac{{({q}_{1}+{q}_{2})}^{2}}{4{R}^{2}}-\frac{{R}^{2}{({q}_{1}-{q}_{2})}^{2}}{4}\right]$$

where $R=\exp (|\tau |)$. Equation (8) means the squeezing effect appears in the diagonal direction. A rotation of $({q}_{1},{q}_{2})$ by angle ${\theta }_{0}$,

7 $$\left[\begin{array}{c}{Q}_{1}\\ {Q}_{2}\end{array}\right]=\left[\begin{array}{cc}\cos {\theta }_{0} & -\,\sin {\theta }_{0}\\ \sin {\theta }_{0} & \cos {\theta }_{0}\end{array}\right]\left[\begin{array}{c}{q}_{1}/\sqrt{2}\\ {q}_{2}/\sqrt{2}\end{array}\right]$$

changes the squeezing direction. We plot the probability ${|{\varPsi }_{sq}({Q}_{1},{Q}_{2})|}^{2}$ with ${\theta }_{0}=\pi /4$, i.e., ${\varPsi }_{sq}({q}_{1},{q}_{2})=\frac{1}{\sqrt{\pi }}\exp [-{q}_{1}^{2}/(4{R}^{2})-({R}^{2}{q}_{2}^{2})/4]$, in the left subfigure of Fig. 2b.

### Experiment

(1) Numerical masks. Two liquid-crystal spatial light modulators, SLM1 and SLM2, are exploited to generate and detect the structured light analogies of quantum squeezed states. Liquid-crystal SLM is an opto-electrical device for phase modulation via regulating the extraordinary refractive index of liquid crystal cells. This method requires numerical masks, which are computer-generated holograms. The masks could be expressed as^56^:

8 $${f}_{mask}=\exp \{i{J}_{1}^{-1}[CA(x,y)]\sin [P(x,y)+2\pi ({u}_{x}x+{v}_{y}y)]\}$$

where $C=0.5819$ is a constant, ${J}_{1}^{-1}[\cdot ]$ is the inverse of the 1st-order Bessel function, $A(x,y)$ and $P(x,y)$ are the target complex amplitude and phase, $({u}_{x},{v}_{y})$ is the spatial frequency coordinate.

**Fig. 6:**
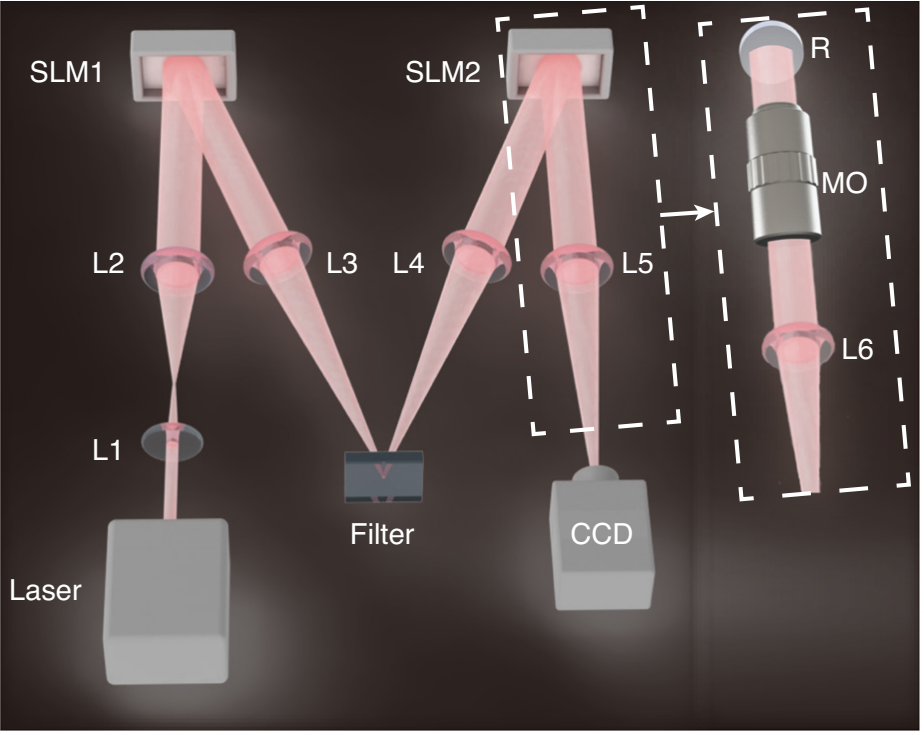
Schematic diagram of the experiment. SLM: liquid-crystal spatial light modulator; L1 to L7: lenses, where the focal lengths of L1 and L2 are 20 mm and 120 mm, composing a $4f$ system with 6× magnification, while the focal length of L3 and L6 is 200 mm; Filter: aperture and reflector; CCD charge-coupled device camera, R reflector, MO microscope objective

(2) The generation and detection of classical squeezed light. The experimental setup is illustrated in Fig. 6. Lenses L1 and L2 expand and collimate the laser beam, which is then modulated by SLM1. The generated several order components go through a $4f$ system (L3 and L4) and are filtered in the Fourier plane to extract the +1st-order component (Filter). The focal plane of L4 corresponds to $z=0$ in Fig. 4. The filtered beam is the structured light analogy of the quantum squeezed state. SLM2, used to detect the modal spectrum, is loaded with the mask $\langle {u}_{n,m}|$, which is the conjugate of the $|{u}_{n,m}\rangle$. The demodulated light is Fourier transformed by a lens L5 (labeled as “FT” in Fig. 3d). The modal spectrum information ${|\langle {u}_{n,m}|\psi \rangle |}^{2}$ is carried by the intensity of the +1st-order component, which is detected by a CCD camera. The complete modal spectrum is obtained by changing the mask (indices of $\langle {u}_{n,m}|$) and recording corresponding intensities.

States $|{u}_{n,m}\rangle$ and $|{u}_{\tau }\rangle$ describe the transverse pattern of structured light, and the propagation factor should be accounted for a real beam, represented as ${U}_{n,m}(x,y,z)$ and ${u}_{\tau }(x,y,z)$, respectively. Therefore, in the experiment, we detect ${|{c}_{n,m}|}^{2}=\iint {U}_{n,m}^{\ast }(x,y,z){u}_{\tau }(x,y,z)dxdy$. The value of ${|{c}_{n,m}|}^{2}$ is independent of $z$. In this work, we detect the modal spectrum at the $z=0$ plane.

To correct the aberrations in the experiment, we use the Laguerre-Gaussian mode^57^. If not corrected, the Laguerre-Gaussian mode shows an elliptical or near Hermite Laguerre-Gaussian mode shape. We use the Zernike polynomials to load a correction phase on the SLM and tune their parameters until the Laguerre-Gaussian mode shows a circular pattern, i.e., an ideal vortex mode.

(3) Tight focusing (experiment). For this experiment, we replace the detection part (left dashed box in Fig. 6) by a microscope objective (right dashed box). Some additional optical components, not shown here, are used to couple the generated beams to the microscope objective. These components are shown in the full detail diagram in Supplementary Material. The microscope objective tightly focuses the paraxial beams at the reflector R. L6 and the microscope objective assembles a $4f$ system with 60× magnification. The physical pixel size of the CCD camera is 6.5 μm. The images of tightly focused beams in Fig. 5 are recorded on a very small area (65 × 65 pixels), and we selected the area with the least noise. Therefore, the images in Fig. 5 look cleaner than in Fig. 4, where for the latter one, the large area (1000 × 1000 pixels) is more susceptible to camera noise and ambient scattering.

### Tight focusing (simulation)

**Fig. 7:**
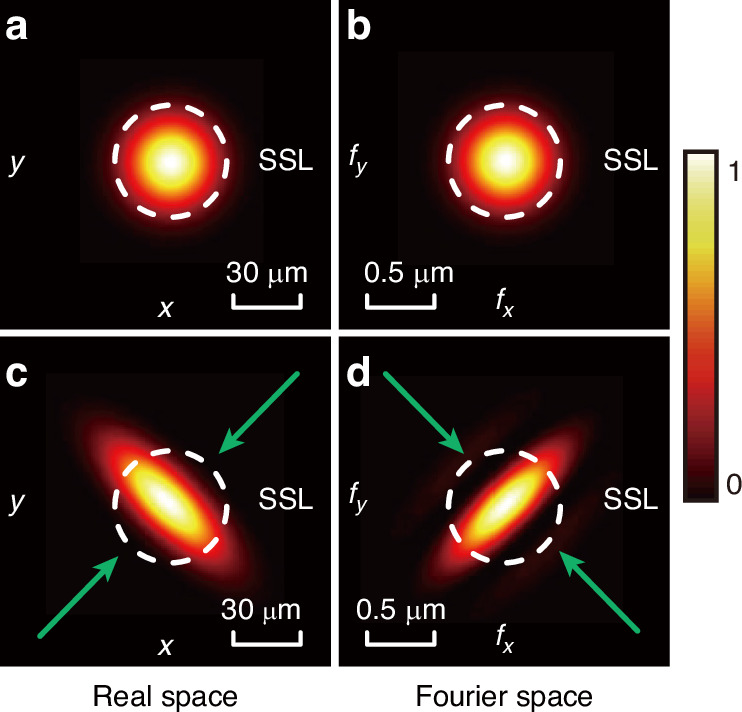
Simulation of the focusing process. The intensity distributions of the fundamental Gaussian beam in **a** real space $(x,y)$ and **b** Fourier space $({f}_{x},{f}_{y})$. The intensity distributions of the classical squeezed structured light in **c** real space and **d** Fourier space. The white dashed circles mark the SSL while the green arrows mark the squeezing direction. The Fourier transform is performed via a focusing lens with 60× magnification

The focused electro-magnetic field could be calculated via non-paraxial vector diffraction theory based on the Debye approximation, which is expressed in angular spectrum representation as^58^:

9 $${\bf{E}}(\rho ,{\varphi }_{2},z)=\frac{ikf{e}^{-ikf}}{2\pi }{\int }_{\!0}^{{\vartheta }_{\max }}{\int }_{\!0}^{2\pi }{\tilde{{\bf{E}}}}_{input}(\vartheta ,{\varphi }_{1}){e}^{ikz\cos \vartheta }{e}^{ik\rho \sin \vartheta \cos ({\varphi }_{1}-{\varphi }_{2})}\sin \vartheta d\vartheta d{\varphi }_{1}$$

where $f$ is the focal length, $k$ is the wavenumber, $\rho$ and ${\varphi }_{2}$ represent the radial and azimuthal coordinates in the focused plane, ${\varphi }_{1}$ is the azimuthal coordinate in the input plane, $z$ is the longitudinal distance between the focused plane and the input plane, $\vartheta$ is the incident angle where the maximum incident angle ${\vartheta }_{\max }$ is determined from the ${\rm{NA}}$ as ${\rm{NA}}={n}_{1}\,\sin ({\vartheta }_{\max })$, ${n}_{1}$ and ${n}_{2}$ are the refractive indices of media in the object and image space. A large numerical aperture lens modulates the input electric field ${{\bf{E}}}_{input}$ as^58^:

10 $$\begin{array}{l}{\tilde{{\bf{E}}}}_{input}(\vartheta ,{\varphi }_{1})=\\ {t}^{s}(\vartheta ){{\bf{E}}}_{input}\cdot \left(\begin{array}{c}-\,\sin ({\varphi }_{1})\\ \cos ({\varphi }_{1})\\ 0\end{array}\right)\left(\begin{array}{c}-\,\sin ({\varphi }_{1})\\ \cos ({\varphi }_{1})\\ 0\end{array}\right)\sqrt{\frac{{n}_{1}}{{n}_{2}}}\sqrt{\cos \vartheta }+\\ {t}^{p}(\vartheta ){{\bf{E}}}_{input}\cdot \left(\begin{array}{c}\cos ({\varphi }_{1})\\ \sin ({\varphi }_{1})\\ 0\end{array}\right)\left(\begin{array}{c}\cos ({\varphi }_{1})\cos (\vartheta )\\ \sin ({\varphi }_{1})\cos (\vartheta )\\ -\,\sin (\vartheta )\end{array}\right)\sqrt{\frac{{n}_{1}}{{n}_{2}}}\sqrt{\cos \vartheta }\end{array}$$

where ${t}^{s}(\vartheta )$ and ${t}^{p}(\vartheta )$ are transmission coefficients of the interface at the focal plane for $s-$ and $p-$ polarized beam, respectively. Based on Eq. (11) and Eq. (12), it is possible to calculate optical fields at any position behind the focal lens. The simulation of the classical structured light in real space (before focusing) and Fourier space (after focusing) is shown in Fig. 7.

We note that the focusing process is different from the squeezing process, where the latter is achieved by adding higher-order modes. Also, a strongly focused beam could have an elongated shape in the direction of polarization^58^, which is not the case in this work (the focused Gaussian beams retain a good circular shape, as shown in Fig. 5a and Fig. 7b).

### Correlation coefficient

The correlation coefficient $cor$ is defined as^59^:

11 $$cor=Cov(C,D)/\sqrt{Var[C]Var[D]}$$

where $C$ and $D$ are the modal spectra for simulation and experimental results, $Cov(C,D)$ is the covariance between the $C$ and $D$, $Var[\cdot ]$ is the variance, respectively.

### Fidelity

Fidelity, or distance between two states, is evaluated as^32^:

12 $$F({\psi }_{1},{\psi }_{2})={|\langle {\psi }_{1}|{\psi }_{2}\rangle |}^{2}=\sum _{n,m}|{c}_{n,m}^{\ast }{d}_{n,m}|$$

where $$|{\psi }_{1}\rangle =\sum _{n,m}{c}_{n,m}|n,m\rangle$$, $$|{\psi }_{2}\rangle =\sum _{n,m}{d}_{n,m}|n,m\rangle$$.

## Supplementary information

Supplementary Material

## Data Availability

All data needed to evaluate the conclusions in the paper are present in the paper and supplementary material. Additional data related to this paper may be requested from the authors.

## References

[1] Forbes, A., de Oliveira, M. & Dennis, M. R. Structured light. *Nat. Photonics* **15**, 253–262 (2021).

[2] He, C., Shen, Y. J. & Forbes, A. Towards higher-dimensional structured light. *Light Sci. Appl.* **11**, 205 (2022). 35790711 10.1038/s41377-022-00897-3 PMC9256673

[3] Wan, Z. S. et al. Ultra-degree-of-freedom structured light for ultracapacity information carriers. *ACS Photonics* **10**, 2149–2164 (2023).

[4] Li, C. et al. Arbitrarily structured quantum emission with a multifunctional metalens. *eLight* **3**, 19 (2023).

[5] Wang, S. et al. Flexible generation of structured terahertz fields via programmable exchange-biased spintronic emitters. *eLight* **4**, 11 (2024).

[6] Schott, G. A. Wave mechanics and classical mechanics and electrodynamics. *Nature* **119**, 820–822 (1927).

[7] Qian, X. F. et al. Shifting the quantum-classical boundary: theory and experiment for statistically classical optical fields. *Optica* **2**, 611–615 (2015).

[8] Nape, I. et al. Quantum structured light in high dimensions. *APL Photonics* **8**, 051101 (2023).

[9] Liu, S. L. et al. Classical analogy of a cat state using vortex light. *Commun. Phys.* **2**, 75 (2019).

[10] Schine, N. et al. Synthetic Landau levels for photons. *Nature* **534**, 671–675 (2016). 27281214 10.1038/nature17943

[11] Clark, L. W. et al. Observation of Laughlin states made of light. *Nature* **582**, 41–45 (2020). 32494082 10.1038/s41586-020-2318-5

[12] Corman, L. Light turned into exotic Laughlin matter. *Nature* **582**, 37–38 (2020). 32494075 10.1038/d41586-020-01567-3

[13] Situ, G. H. & Fleischer, J. W. Dynamics of the Berezinskii–Kosterlitz–Thouless transition in a photon fluid. *Nat. Photonics* **14**, 517–522 (2020).

[14] Shen, Y. J. et al. Structured ray-wave vector vortex beams in multiple degrees of freedom from a laser. *Optica* **7**, 820–831 (2020).

[15] Shen, Y. J. et al. Creation and control of high-dimensional multi-partite classically entangled light. *Light Sci. Appl.* **10**, 50 (2021). 33686054 10.1038/s41377-021-00493-x PMC7940607

[16] Guzman-Silva, D. et al. Demonstration of local teleportation using classical entanglement. *Laser Photonics Rev.* **10**, 317–321 (2016).

[17] Ndagano, B. et al. Characterizing quantum channels with non-separable states of classical light. *Nat. Phys.* **13**, 397–402 (2017).

[18] Zhu, Z. Y. et al. Compensation-free high-dimensional free-space optical communication using turbulence-resilient vector beams. *Nat. Commun.* **12**, 1666 (2021). 33712593 10.1038/s41467-021-21793-1 PMC7955115

[19] Wan, Z. S. et al. Divergence-degenerate spatial multiplexing towards future ultrahigh capacity, low error-rate optical communications. *Light Sci. Appl.* **11**, 144 (2022). 35585043 10.1038/s41377-022-00834-4 PMC9117247

[20] Forbes, A., Aiello, A. & Ndagano, B. Classically entangled light. *Prog. Opt.* **64**, 99–153 (2019).

[21] Shen, Y. J. & Rosales-Guzmán, C. Nonseparable states of light: from quantum to classical. *Laser Photonics Rev.* **16**, 2100533 (2022).

[22] Couteau, C. et al. Applications of single photons to quantum communication and computing. *Nat. Rev. Phys.* **5**, 326–338 (2023).

[23] Couteau, C. et al. Applications of single photons in quantum metrology, biology and the foundations of quantum physics. *Nat. Rev. Phys.* **5**, 354–363 (2023).

[24] Aasi, J. et al. Enhanced sensitivity of the LIGO gravitational wave detector by using squeezed states of light. *Nat. Photonics* **7**, 613–619 (2013).

[25] Casacio, C. A. et al. Quantum-enhanced nonlinear microscopy. *Nature* **594**, 201–206 (2021). 34108694 10.1038/s41586-021-03528-w

[26] Malia, B. K. et al. Distributed quantum sensing with mode-entangled spin-squeezed atomic states. *Nature* **612**, 661–665 (2022). 36418400 10.1038/s41586-022-05363-z

[27] Yu, H. C. et al. Quantum correlations between light and the kilogram-mass mirrors of LIGO. *Nature* **583**, 43–47 (2020). 32612226 10.1038/s41586-020-2420-8

[28] Kolobov, M. I. The spatial behavior of nonclassical light. *Rev. Mod. Phys.* **71**, 1539–1589 (1999).

[29] Zheludev, N. I. & Yuan, G. H. Optical superoscillation technologies beyond the diffraction limit. *Nat. Rev. Phys.* **4**, 16–32 (2022).

[30] Zheludev, N. I. What diffraction limit? *Nat. Mater.* **7**, 420–422 (2008). 18497841 10.1038/nmat2163

[31] Chao, P. N. et al. Physical limits in electromagnetism. *Nat. Rev. Phys.* **4**, 543–559 (2022).

[32] Dragoman, D. & Dragoman, M. In *Quantum-Classical Analogies* (eds Dragoman, D. & Dragoman, M.) 1–7 (Springer, 2004).

[33] Gerry, C. & Knight, P. *Introductory Quantum Optics* (Cambridge University Press, 2004).

[34] Nienhuis, G. & Allen, L. Paraxial wave optics and harmonic oscillators. *Phys. Rev. A* **48**, 656–665 (1993). 9909640 10.1103/physreva.48.656

[35] Gbur, G. J. *Mathematical Methods for Optical Physics and Engineering* (Cambridge University Press, 2011).

[36] Lvovsky, A. I. In *Photonics: Scientific Foundations, Technology and Applications* (ed. Andrews, D. L.) 121–163 (John Wiley & Sons, Inc, 2015).

[37] Schleich, W. P. In *Quantum Optics in Phase Space* (ed. Schleich, W. P.) 99–151 (Wiley-VCH Verlag Berlin GmbH, 2001).

[38] Harder, G. et al. Single-mode parametric-down-conversion states with 50 photons as a source for mesoscopic quantum Optics. *Phys. Rev. Lett.* **116**, 143601 (2016). 27104708 10.1103/PhysRevLett.116.143601

[39] Chen, G., Wen, Z. Q. & Qiu, C. W. Superoscillation: from physics to optical applications. *Light Sci. Appl.* **8**, 56 (2019). 31231522 10.1038/s41377-019-0163-9 PMC6560133

[40] Yuan, G. H. & Zheludev, N. I. Detecting nanometric displacements with optical ruler metrology. *Science* **364**, 771–775 (2019). 31072905 10.1126/science.aaw7840

[41] Beck, M. Introductory quantum optics. *Am. J. Phys.* **73**, 1197–1198 (2005).

[42] Zhao, Y. J. et al. Creation of Greenberger-Horne-Zeilinger states with thousands of atoms by entanglement amplification. *npj Quantum Inf.* **7**, 24 (2021).

[43] Zhang, K. et al. Superoscillation focusing with suppressed sidebands by destructive interference. *Opt. Express* **30**, 43127–43142 (2022). 36523018 10.1364/OE.474346

[44] Phillips, R. L. & Andrews, L. C. Spot size and divergence for Laguerre Gaussian beams of any order. *Appl. Opt.* **22**, 643–644 (1983). 18195843 10.1364/ao.22.000643

[45] Inavalli, V. V. G. K. et al. A super-resolution platform for correlative live single-molecule imaging and STED microscopy. *Nat. Methods* **16**, 1263–1268 (2019). 31636458 10.1038/s41592-019-0611-8

[46] Rogers, E. T. F. et al. A super-oscillatory lens optical microscope for subwavelength imaging. *Nat. Mater.* **11**, 432–435 (2012). 22447113 10.1038/nmat3280

[47] Ouyang, W. Q. et al. Ultrafast 3D nanofabrication via digital holography. *Nat. Commun.* **14**, 1716 (2023). 36973254 10.1038/s41467-023-37163-y PMC10043265

[48] Lukosz, W. Optical systems with resolving powers exceeding the classical limit. *J. Opt. Soc. Am.* **56**, 1463–1471 (1966).

[49] Zivari, A. et al. Non-classical mechanical states guided in a phononic waveguide. *Nat. Phys.* **18**, 789–793 (2022).

[50] Arwas, G. et al. Anyonic-parity-time symmetry in complex-coupled lasers. *Sci. Adv.* **8**, eabm7454 (2022). 35648848 10.1126/sciadv.abm7454 PMC9159572

[51] McMahon, P. L. The physics of optical computing. *Nat. Rev. Phys.* **5**, 717–734 (2023).

[52] Meng, Y. et al. Optical meta-waveguides for integrated photonics and beyond. *Light Sci. Appl.* **10**, 235 (2021). 34811345 10.1038/s41377-021-00655-x PMC8608813

[53] Hiekkamäki, M. & Fickler, R. High-dimensional two-photon interference effects in spatial modes. *Phys. Rev. Lett.* **126**, 123601 (2021). 33834827 10.1103/PhysRevLett.126.123601

[54] Vetlugin, A. N. Coherent perfect absorption of quantum light. *Phys. Rev. A* **104**, 013716 (2021).

[55] Wang, X. L. et al. Quantum teleportation of multiple degrees of freedom of a single photon. *Nature* **518**, 516–519 (2015). 25719668 10.1038/nature14246

[56] Arrizón, V. et al. Pixelated phase computer holograms for the accurate encoding of scalar complex fields. *J. Opt. Soc. Am. A* **24**, 3500–3507 (2007). 10.1364/josaa.24.003500 17975577

[57] Scholes, S. et al. Structured light with digital micromirror devices: a guide to best practice. *Opt. Eng.* **59**, 041202 (2019).

[58] Novotny, L. & Hecht, B. *Principles of Nano-Optics* (Cambridge University Press, 2012).

[59] Baykal, Y. Field correlations of a partially coherent optical Gaussian wave in tissue turbulence. *J. Opt. Soc. Am. A* **39**, C6–C11 (2022). 10.1364/JOSAA.470029 36520717

