# Supplementary material for: Structured light analogy of quantum squeezed states
Source: Light Sci Appl. 2024 Oct 21;13:297. doi: 10.1038/s41377-024-01631-x (PMC11491454; doi:10.1038/s41377-024-01631-x)
Supplement: Supplementary file 1 — Supplementary Material [file 41377_2024_1631_MOESM1_ESM.pdf]

Supplementary Material for: Structured light analogy of quantum squeezed states

Zhaoyang Wang,[1,2,3] Ziyu Zhan,[1,2,3] Anton N. Vetlugin,[4] Jun-Yu Ou,[5] Qiang Liu,[1,2,3,*] Yijie Shen,[4,6,†] and Xing Fu[1,2,3,‡]

[1]*Department of Precision Instrument, Tsinghua University, Beijing 100084, China*
[2]*State Key Laboratory of Precision Space-Time Information Sensing Technology, Beijing 100084, China*
[3]*Key Laboratory of Photonic Control Technology (Tsinghua University), Ministry of Education, Beijing 100084, China*
[4]*Centre for Disruptive Photonic Technologies, School of Physical and Mathematical Sciences & The Photonics Institute, Nanyang Technological University, Singapore 637371, Singapore*
[5]*School of Physics and Astronomy, University of Southampton, Southampton, United Kingdom*
[6]*School of Electrical and Electronic Engineering, Nanyang Technological University, Singapore 639798, Singapore*

(Dated: August 3, 2024)

This document provides supplementary information to the paper titled "Structured light analogy of quantum squeezed states". It includes detailed theoretical analysis and experimental results supporting the main article.

Contents

Theoretical basis: the analogy between number states and Hermite-Gaussian beams 1
Harmonic oscillator and number states 1
Paraxial wave equation and Hermite-Gaussian beams 2
The mathematical analogy between number states and Hermite-Gaussian beams 4

Structured light analogy of squeezed states 5
Structured light analogy of the squeezed vacuum state 5
Structured light analogy of squeezed number state 7
The wavefunction of quantum two-mode states 8
Q function for quantum single-mode states 9
Astigmatic Gaussian beams 11

The generation and detection of structured light analogy of squeezed states 11

Tightly focusing results 12

Acknowledgment 13

References 13

THEORETICAL BASIS: THE ANALOGY BETWEEN NUMBER STATES AND HERMITE-GAUSSIAN BEAMS

The basic idea for the structured light analogy of quantum states is originated from the mathematical analogy between number states and Hermite-Gaussian (HG) beams.

Harmonic oscillator and number states

Number states are defined from the quantum harmonic oscillator. The time-dependent Schrödinger equation with the harmonic operator is defined as [1]:

$$\mathrm{i}\hbar\frac{\partial}{\partial t}\left|\Psi\right\rangle = \hat{\mathcal{H}}\left|\Psi\right\rangle \tag{S.1}$$

where $\hat{\mathcal{H}}$ is the harmonic operator, $\hbar$ is the reduced Planck constant, $|\Psi\rangle$ represents the eigenstate of the harmonic operator, $\Psi(\mathbf{r},t)$ represents the wave function, $|\Psi(\mathbf{r},t)|^2$ is the probability distribution, $\mathbf{r}$ and t represent position and time, respectively. For the harmonic operator $\hat{\mathcal{H}}$ without explicit t, the Eq. (S.1) could be separated as [1]:

$$\mathrm{i}\hbar\frac{\mathrm{d}}{\mathrm{d}t}T(t) = ET(t) \tag{S.2}$$

$$\hat{\mathcal{H}}\left|\psi\right\rangle = E\left|\psi\right\rangle \tag{S.3}$$

where E is a constant corresponding to the eigenenergy, $|\Psi\rangle = T(t)\,|\psi\rangle$. The solution of Eq. (S.2) could be expressed as $T(t) = A\exp(-\mathrm{i}Et/\hbar)$, where A is a constant. Eq. (S.3) is the equation describing the time-independent harmonic oscillator. In fact, the eigenstate $|\psi\rangle$ of Eq. (S.3) is the number state.

The quantification of electromagnetic fields. As for a single-mode quantified electromagnetic field, its harmonic operator is defined as [1]:

$$\hat{\mathcal{H}} = \frac{1}{2}\left(\hat{p}^2 + \omega^2\hat{q}^2\right) \tag{S.4}$$

where $\hat{p}$ and $\hat{q}$ are Hermitian, $[\hat{q},\hat{p}] = \mathrm{i}\hbar$, ω is the eigenfrequency of the electromagnetic field, and the annihilation ($\hat{a}$) and creation ($\hat{a}^\dagger$) operators could be expressed as:

$$\hat{a} = (2\hbar\omega)^{-1/2}\left(\omega\hat{q} + \mathrm{i}\hat{p}\right) \tag{S.5}$$

$$\hat{a}^{\dagger} = (2\hbar\omega)^{-1/2} (\omega\hat{q} - \mathrm{i}\hat{p}) \tag{S.6}$$

where $\left[\hat{a}, \hat{a}^{\dagger}\right] = 1$. The corresponding electric and magnetic field operators are:

$$\hat{E}_x(z,t) = \varepsilon_0 \left(\hat{a} + \hat{a}^{\dagger}\right) \sin(kz) \tag{S.7}$$

$$\hat{B}_y(z,t) = \beta_0 \left(\hat{a} - \hat{a}^{\dagger}\right) \cos(kz) \tag{S.8}$$

where $\varepsilon_0 = (\hbar\omega/\epsilon_0 V)^{1/2}$ and $\beta_0 = (\mu_0/k)\left(\epsilon_0\hbar\omega^3/V\right)$, V is the effective volume of the cavity, $k = \omega/c$ is the wavenumber, c is the light speed, ϵ_0 is the capacitance, μ_0 is the magnetic permeability. The harmonic operator could also take the form as $\hat{\mathcal{H}} = \hbar\omega\left(\hat{a}^{\dagger}\hat{a} + 1/2\right)$.

The operator product $\hat{a}^{\dagger}\hat{a}$ is the number operator that $\hat{a}^{\dagger}\hat{a}\left|n\right\rangle = n\left|n\right\rangle$. The eigenstate of a single-mode field is denoted as $\left|n\right\rangle$, $\hat{\mathcal{H}}\left|n\right\rangle = E_n\left|n\right\rangle$, where $E_n = \hbar\omega\,(n + 1/2)$. The annihilation and creation processes could be described as $\hat{a}|n\rangle = \sqrt{n}|n-1\rangle$ and $\hat{a}^{\dagger}|n\rangle = \sqrt{n+1}|n+1\rangle$, respectively.

As for a two-mode quantified electromagnetic field, its harmonic operator is defined as [1]:

$$\hat{\mathcal{H}} = \frac{1}{2}\sum_j \left(\hat{p}_j^2 + \omega^2\hat{q}_j^2\right) \tag{S.9}$$

where $j = 1,2$ represent distinct modes. The commutation relation between $\hat{p}_j$ and $\hat{q}_j$ is denoted as $[\hat{q}_j, \hat{p}_j] = \mathrm{i}\hbar$. The harmonic operator could also be written with ladder operators as $\hat{\mathcal{H}} = \sum_{j=1,2} \hbar\omega\left(\hat{a}_j^{\dagger}\hat{a}_j + 1/2\right)$, where ($\hat{a}_j$, $\hat{a}_j^{\dagger}$) are the ladder operators for distinct modes, $\hat{a}_j = (2\hbar\omega)^{-1/2}(\omega\hat{q}_j + \mathrm{i}\hat{p}_j)$, $\hat{a}_j^{\dagger} = (2\hbar\omega)^{-1/2}(\omega\hat{q}_j - \mathrm{i}\hat{p}_j)$. The eigenstate of a two-mode field is denoted as $\left|n,m\right\rangle$. Thus, $\hat{\mathcal{H}}\left|n,m\right\rangle = E_{n,m}\left|n,m\right\rangle$, where $E_{n,m} = \hbar\omega\,(n+m+1)$.

The expressions in the main text are written in dimensionless forms (assuming $\hbar = 1$, $\omega = 1$). The dimensionless harmonic operator is:

$$\hat{\mathcal{H}} = \sum_{j=1,2}\left(\hat{p}_j^2 + \hat{q}_j^2\right)/2 = \sum_{j=1,2}\left(\hat{a}_j^{\dagger}\hat{a}_j + 1/2\right) \tag{S.10}$$

where $[\hat{q}_j, \hat{p}_j] = \mathrm{i}$, $E_{n,m} = n + m + 1$.

The annihilation and creation processes could be described as $\hat{a}_1|n,m\rangle = \sqrt{n}|n-1,m\rangle$, $\hat{a}_1^{\dagger}|n,m\rangle = \sqrt{n+1}|n+1,m\rangle$, $\hat{a}_2|n,m\rangle = \sqrt{m}|n,m-1\rangle$, $\hat{a}_2^{\dagger}|n,m\rangle = \sqrt{m+1}|n,m+1\rangle$, respectively. The operator product $\sum_{j=1,2}\hat{a}_j^{\dagger}\hat{a}_j$ is the number operator that $\sum_{j=1,2}\hat{a}_j^{\dagger}\hat{a}_j\left|n,m\right\rangle = (n+m)\left|n,m\right\rangle$.

Number states in coordinate representation. The time-independent equation of the harmonic oscillator in coordinate representation could be expressed as:

$$\left[-\frac{\hbar^2}{2}\nabla_{\mathrm{T}}^2 + \frac{1}{2}(\omega^2x^2 + \omega^2y^2)\right]\psi(x,y) = E_{n,m}\psi(x,y) \tag{S.11}$$

where $\left[-\hbar^2\nabla_{\mathrm{T}}^2 + \omega^2(x^2+y^2)\right]/2$ is the operator of the time-independent harmonic oscillator in coordinate representation, $\psi(x,y)$ is the wave function of two-mode number states, $\nabla_{\mathrm{T}}^2 = \frac{\partial^2}{\partial x^2} + \frac{\partial^2}{\partial y^2}$. After separating $\psi(x,y) = \psi(x)\psi(y)$, the Eq. (S.11) could be separated as:

$$\left(-\frac{\hbar^2}{2}\frac{\mathrm{d}^2}{\mathrm{d}x^2} + \frac{1}{2}\omega^2x^2\right)\psi(x) = E_n\psi(x) \tag{S.12}$$

$$\left(-\frac{\hbar^2}{2}\frac{\mathrm{d}^2}{\mathrm{d}y^2} + \frac{1}{2}\omega^2y^2\right)\psi(y) = E_m\psi(y) \tag{S.13}$$

where $E = E_n + E_m$. Since the Eq. (S.12) has the same mathematical form as the Eq. (S.13), we take the Eq. (S.12) as the example, which could be simplified by $x' = \mu x$, $\mu^2 = \omega/\hbar$, $\chi = 2E_n/(\hbar\omega)$ as:

$$\frac{\mathrm{d}^2\psi(x')}{\mathrm{d}x'^2} + \chi\psi(x') - x'^2\psi(x') = 0 \tag{S.14}$$

where $\psi(x')$ could be constructed in Gaussian form as $\psi(x') = \exp(-x'^2/2)\zeta(x')$, and then substituted into Eq. (S.14) as [1]:

$$\frac{\mathrm{d}^2\zeta(x')}{\mathrm{d}x'^2} - 2x'\frac{\mathrm{d}\zeta(x')}{\mathrm{d}x'} + (\chi - 1)\zeta(x') = 0 \tag{S.15}$$

where $\chi = 2n + 1$, n is a non-negative integer. The Eq. (S.15) has the Hermite equation form, and the solution $\zeta(x')$ has the Hermite function form as $H_n(x')$. Thus, the solution of Eq. (S.14) is the HG function as:

$$\psi_n(x') = \frac{1}{\sqrt{2^n n!\sqrt{\pi}}}H_n(x')\mathrm{e}^{-x'^2/2} \tag{S.16}$$

where $E_n = (n + 1/2)\hbar\omega$ is the eigenvalue E_n of Eq. (S.14). The solution Eq. (S.16) could be written in x parameter as:

$$\psi_n(x) = \frac{(\omega/\hbar)^{1/4}}{\sqrt{2^n n!\sqrt{\pi}}}\exp\left(-\frac{\omega x^2}{2\hbar}\right)H_n\left(\sqrt{\frac{\omega}{\hbar}}x\right) \tag{S.17}$$

where $(\omega/\hbar)^{1/4}$ is the normalization factor. The solution for Eq. (S.13) could be expressed as $\psi_m(y')$ or $\psi_m(y)$. Its eigenvalue is $E_m = (m + 1/2)\hbar\omega$. The two-mode number state $|n,m\rangle$ in coordinate representation is a HG function that $\psi_{n,m}(x,y) = \psi_n(x)\psi_m(y)$.

Paraxial wave equation and Hermite-Gaussian beams

The analogy between paraxial modes and quantum harmonic oscillators is the base of quantum-classical analogy [2].

Paraxial wave equation. The paraxial structured light obeys the PWE as [3]:

$$\nabla_{\mathrm{T}}^2U(x,y,z) + 2\mathrm{i}k\frac{\partial U(x,y,z)}{\partial z} = 0 \tag{S.18}$$

where $U(x,y,z)$ is the complex amplitude distribution of paraxial structured light, $|U(x,y,z)|^2$ is the intensity, $k=2\pi/\lambda$ is the wavenumber, λ is the wavelength. There is a Gaussian function solution for PWE, noted as $U_0(x,y,z)$ [3]:

$$U_0(x,y,z)=\frac{A_0}{w(z)}\mathrm{e}^{\mathrm{i}k[z+\frac{x^2+y^2}{2R(z)}]}\mathrm{e}^{-\mathrm{i}\tan^{-1}\left(\frac{z}{z_R}\right)}\mathrm{e}^{-\frac{x^2+y^2}{w^2(z)}} \tag{S.19}$$

where $R(z)=z+z_R^2/z$ is the wavefront curvature, $w(z)=w_0\sqrt{1+(z/z_R)^2}$ is the beam waist parameter, $w_0=\sqrt{\lambda z_R/\pi}$, z_R is the Rayleigh range. Based on the solution form of Eq. (S.19), we consider a high order form as $U(x,y,z)=U_0(x,y,z)f[\sqrt{2}x/w(z)]g[\sqrt{2}y/w(z)]\exp[\mathrm{i}\vartheta(z)]$, which is substituted into Eq. (S.18) as:

$$\begin{aligned}&gU_0\frac{\partial^2 f}{\partial x^2}+2g\frac{\partial U_0}{\partial x}\frac{\partial f}{\partial x}+fU_0\frac{\partial^2 g}{\partial y^2}+2f\frac{\partial U_0}{\partial y}\frac{\partial g}{\partial y}+\\&2\mathrm{i}k[gU_0\frac{\partial f}{\partial z}+fU_0\frac{\partial g}{\partial z}]+fg\{\frac{\partial^2 U_0}{\partial x^2}+\frac{\partial^2 U_0}{\partial y^2}+2\mathrm{i}k\frac{\partial U_0}{\partial z}\}\\&-2kfgU_0\frac{\mathrm{d}\vartheta(z)}{\mathrm{d}z}=0\end{aligned} \tag{S.20}$$

where we note $g[\sqrt{2}y/w(z)]$ as g, $f[\sqrt{2}x/w(z)]$ as f, and $U_0(x,y,z)$ as U_0 for simplicity, $\partial^2U_0/\partial x^2+\partial^2U_0/\partial y^2+2\mathrm{i}k\partial U_0/\partial z=0$. By substituting $\xi=\sqrt{2}x/w(z)$, $\eta=\sqrt{2}y/w(z)$ into Eq. (S.20), we obtain:

$$\begin{aligned}&\frac{1}{f}\frac{\partial^2 f}{\partial\xi^2}+\left(\frac{\mathrm{i}kw^2(z)}{R(z)}-2\right)\frac{\xi}{f}\frac{\partial f}{\partial\xi}+\left(\frac{\mathrm{i}kw^2(z)}{R(z)}-2\right)\frac{\eta}{g}\frac{\partial g}{\partial\eta}\\&+\frac{1}{g}\frac{\partial^2 g}{\partial\eta^2}-\mathrm{i}kw(z)\frac{\mathrm{d}w(z)}{\mathrm{d}z}\left(\frac{\xi}{f}\frac{\partial f}{\partial\xi}+\frac{\eta}{g}\frac{\partial g}{\partial\eta}\right)-kw^2(z)\frac{\mathrm{d}\vartheta(z)}{\mathrm{d}z}\\&=0\end{aligned} \tag{S.21}$$

where

$$w(z)\frac{\mathrm{d}w(z)}{\mathrm{d}z}=w_0^2\frac{z}{z_R^2}=\frac{w^2(z)}{R(z)} \tag{S.22}$$

$$\frac{\partial}{\partial x}=\frac{\sqrt{2}}{w(z)}\frac{\partial}{\partial\xi},\frac{\partial}{\partial y}=\frac{\sqrt{2}}{w(z)}\frac{\partial}{\partial\eta} \tag{S.23}$$

$$\frac{\partial f}{\partial z}=\frac{\partial\xi}{\partial z}\frac{\partial f}{\partial\xi}=-\frac{\xi}{w(z)}\frac{\mathrm{d}w(z)}{\mathrm{d}z}\frac{\partial f}{\partial\xi} \tag{S.24}$$

$$\frac{\partial g}{\partial z}=\frac{\partial\eta}{\partial z}\frac{\partial g}{\partial\eta}=-\frac{\eta}{w(z)}\frac{\mathrm{d}w(z)}{\mathrm{d}z}\frac{\partial g}{\partial\eta} \tag{S.25}$$

The Eq. (S.20) could be further simplified as:

$$\frac{1}{f}\frac{\partial^2 f}{\partial\xi^2}-2\frac{\xi}{f}\frac{\partial f}{\partial\xi}+\frac{1}{g}\frac{\partial^2 g}{\partial\eta^2}-2\frac{\eta}{g}\frac{\partial g}{\partial\eta}-kw^2(z)\frac{\mathrm{d}\vartheta(z)}{\mathrm{d}z}=0 \tag{S.26}$$

The separated set of Eq. (S.26) could be expressed as [3]:

$$\frac{\partial^2 f}{\partial\xi^2}-2\xi\frac{\partial f}{\partial\xi}+2nf=0 \tag{S.27}$$

$$\frac{\partial^2 g}{\partial\eta^2}-2\eta\frac{\partial g}{\partial\eta}+2mg=0 \tag{S.28}$$

$$\frac{\mathrm{d}\vartheta(z)}{\mathrm{d}z}=-\frac{2(n+m)}{kw^2(z)} \tag{S.29}$$

where Eq. (S.27) and Eq. (S.28) have the Hermite equation forms. Thus, their solutions are Hermite functions as $f(\xi)=H_n(\xi)$ and $g(\eta)=H_m(\eta)$. As for Eq. (S.29), it can be directly integrated as $\vartheta(z)=-(n+m)\tan^{-1}(z/z_R)$.

According to the orthogonality condition, the HG beams could be expressed as:

$$\begin{aligned}&U_{n,m}(x,y,z)=\frac{1}{\sqrt{\pi 2^{n+m-1}n!m!}w(z)}H_n(\frac{\sqrt{2}x}{w(z)})H_m(\frac{\sqrt{2}y}{w(z)})\\&\mathrm{e}^{\mathrm{i}k\left(z+\frac{x^2+y^2}{2R(z)}\right)-\mathrm{i}(n+m+1)\tan^{-1}\left(\frac{z}{z_R}\right)}\mathrm{e}^{-\frac{x^2+y^2}{w(z)^2}}\end{aligned} \tag{S.30}$$

which could be noted as:

$$U_{n,m}(x,y,z)=u_{n,m}(\xi,\eta)\phi(z) \tag{S.31}$$

where the $u_{n,m}(\xi,\eta)=u_n(\xi)u_m(\eta)$ describes the transverse spatial modes of HG beams, and the other part $\phi(z)$ is the propagation factor [3]:

$$u_n(\xi)=\left(2^n n!\sqrt{\pi}\right)^{-1/2}\mathrm{e}^{-\xi^2/2}H_n(\xi) \tag{S.32}$$

$$u_m(\eta)=\left(2^m m!\sqrt{\pi}\right)^{-1/2}\mathrm{e}^{-\eta^2/2}H_m(\eta) \tag{S.33}$$

$$\phi(z)=\frac{\sqrt{2}}{w(z)}\mathrm{e}^{\left[\mathrm{i}k(z+\frac{x^2+y^2}{2R(z)})-\mathrm{i}(n+m+1)\tan^{-1}(\frac{z}{z_R})\right]} \tag{S.34}$$

It is interesting that Eq. (S.32) and Eq. (S.33) have the same forms as the Eq. (S.16), which unveils that the transverse spatial modes of structured light could be seen as the structured light counterpart of number states, i.e. $|n,m\rangle\leftrightarrow u_{n,m}(\xi,\eta)$. There should exist an equation for the transverse spatial mode $u_{n,m}(\xi,\eta)$, as the structured light counterpart of the time-independent harmonic equation Eq. (S.3), which could be named as "the transverse spatial eigenmode equation".

Transverse eigenmode equation. The expression of HG beams could be rewritten as $U_{n,m}(\xi,\eta,z)=u_{n,m}(\xi,\eta)\phi'(z)\exp\left[\mathrm{i}k\left(x^2+y^2\right)/2/R(z)\right]$, where $\phi'(z)=\phi(z)/\exp\left[\mathrm{i}k\left(x^2+y^2\right)/2/R(z)\right]$, which would be substituted into PWE in Eq. (S.18). Some terms are derived for brevity as:

$$\begin{aligned}&\nabla_{\mathrm{T}}^2U_{n,m}(\xi,\eta,z)=\phi'(z)\nabla_{\mathrm{T}}^2[u_{n,m}(\xi,\eta)\mathrm{e}^{\mathrm{i}k\frac{x^2+y^2}{2R(z)}}]\\&=\phi'(z)\mathrm{e}^{\mathrm{i}k\frac{x^2+y^2}{2R(z)}}\nabla_{\mathrm{T}}^2u_{n,m}(\xi,\eta)+\phi'(z)u_{n,m}(\xi,\eta)\nabla_{\mathrm{T}}^2\mathrm{e}^{\mathrm{i}k\frac{x^2+y^2}{2R(z)}}\\&+2\phi'(z)(\frac{\partial u_{n,m}(\xi,\eta)}{\partial x}\frac{\partial\mathrm{e}^{\mathrm{i}k\frac{x^2+y^2}{2R(z)}}}{\partial x}+\frac{\partial u_{n,m}(\xi,\eta)}{\partial y}\frac{\partial\mathrm{e}^{\mathrm{i}k\frac{x^2+y^2}{2R(z)}}}{\partial y})\end{aligned} \tag{S.35}$$

$$\nabla_{\mathrm{T}}^2 \mathrm{e}^{\mathrm{i}k\frac{x^2+y^2}{2R(z)}} = \left[\frac{2\mathrm{i}k}{R(z)} - \frac{k^2x^2}{R^2(z)}\right]\mathrm{e}^{\mathrm{i}k\frac{x^2+y^2}{2R(z)}} \tag{S.36}$$

$$\frac{\partial u_{n,m}(\xi,\eta)}{\partial x}\frac{\partial \mathrm{e}^{\mathrm{i}k\frac{x^2+y^2}{2R(z)}}}{\partial x} = \frac{\mathrm{i}kx}{R(z)}\frac{\sqrt{2}}{w(z)}\frac{\partial u_{n,m}(\xi,\eta)}{\partial \xi}\mathrm{e}^{\mathrm{i}k\frac{x^2+y^2}{2R(z)}} \tag{S.37}$$

$$\frac{\partial u_{n,m}(\xi,\eta)}{\partial y}\frac{\partial \mathrm{e}^{\mathrm{i}k\frac{x^2+y^2}{2R(z)}}}{\partial y} = \frac{\mathrm{i}ky}{R(z)}\frac{\sqrt{2}}{w(z)}\frac{\partial u_{n,m}(\xi,\eta)}{\partial \eta}\mathrm{e}^{\mathrm{i}k\frac{x^2+y^2}{2R(z)}} \tag{S.38}$$

$$\begin{aligned}\frac{\partial U_{n,m}(\xi,\eta,z)}{\partial z} &= \mathrm{e}^{\mathrm{i}k\frac{x^2+y^2}{2R(z)}}u_{n,m}(\xi,\eta)\frac{\partial \phi'(z)}{\partial z}\\ &+ \phi'(z)u_{n,m}(\xi,\eta)\frac{\partial \mathrm{e}^{\mathrm{i}k\frac{x^2+y^2}{2R(z)}}}{\partial z} + \phi'(z)\mathrm{e}^{\mathrm{i}k\frac{x^2+y^2}{2R(z)}}\frac{\partial u_{n,m}(\xi,\eta)}{\partial z}\end{aligned} \tag{S.39}$$

$$\frac{\partial \mathrm{e}^{\mathrm{i}k\frac{x^2+y^2}{2R(z)}}}{\partial z} = -\mathrm{i}k\frac{x^2+y^2}{2R^2(z)}\frac{\mathrm{d}R(z)}{\mathrm{d}z}\mathrm{e}^{\mathrm{i}k\frac{x^2+y^2}{2R(z)}} \tag{S.40}$$

$$\frac{\partial u_{n,m}(\xi,\eta)}{\partial z} = -\frac{1}{w(z)}\frac{\mathrm{d}w(z)}{\mathrm{d}z}\left(\xi\frac{\partial}{\partial\xi} + \eta\frac{\partial}{\partial\eta}\right)u_{n,m}(\xi,\eta) \tag{S.41}$$

$$\frac{1}{w(z)}\frac{\mathrm{d}w(z)}{\mathrm{d}z} = \frac{1}{R(z)} \tag{S.42}$$

Based on these results, the PWE, after substituting $U_{n,m}(\xi,\eta,z)$ into Eq. (S.18), could be derived as:

$$\begin{aligned}&\phi'(z)\nabla_{\mathrm{T}}^2 u_{n,m}(\xi,\eta) + \phi'(z)u_{n,m}(\xi,\eta)\left[\frac{2\mathrm{i}k}{R(z)} - \frac{k^2(x^2+y^2)}{R^2(z)}\right]\\ &+ \phi'(z)\frac{2\mathrm{i}k}{R(z)}\left[\xi\frac{u_{n,m}(\xi,\eta)}{\xi} + \eta\frac{u_{n,m}(\xi,\eta)}{\eta}\right] +\\ &2\mathrm{i}ku_{n,m}(\xi,\eta)\frac{\partial\phi'(z)}{\partial z} + \frac{k^2(x^2+y^2)}{R^2(z)}u_{n,m}(\xi,\eta)\phi'(z)\frac{\mathrm{d}R(z)}{\mathrm{d}z} -\\ &\phi'(z)\frac{2\mathrm{i}k}{R(z)}\left[\xi\frac{u_{n,m}(\xi,\eta)}{\xi} + \eta\frac{u_{n,m}(\xi,\eta)}{\eta}\right] = 0\end{aligned} \tag{S.43}$$

Some terms could be rewritten as an analytical form as:

$$\frac{k^2}{R^2(z)}\left[\frac{\mathrm{d}R(z)}{\mathrm{d}z} - 1\right] = -\frac{k^2}{R^2(z)}\frac{z_R^2}{z^2} = -\frac{k^2}{z_R^2(1+(z/z_R)^2)} \tag{S.44}$$

Considering $k = 2z_R/w_0^2$:

$$\frac{k^2}{R^2(z)}\left[\frac{\mathrm{d}R(z)}{\mathrm{d}z} - 1\right] = -\frac{4}{w^4(z)} \tag{S.45}$$

then Eq. (S.43) could be rewritten as:

$$\begin{aligned}&\phi'(z)\nabla_{\mathrm{T}}^2 u_{n,m}(\xi,\eta) - \frac{4}{w^4(z)}(x^2+y^2)\phi'(z)u_{n,m}(\xi,\eta)\\ &+ 2\mathrm{i}ku_{n,m}(\xi,\eta)\frac{\mathrm{d}\phi'(z)}{\mathrm{d}z} + \frac{2\mathrm{i}k}{R(z)}\phi'(z)u_{n,m}(\xi,\eta) = 0\end{aligned} \tag{S.46}$$

which can be separated as:

$$\nabla_{\mathrm{T}}^2 u_{n,m}(\xi,\eta) - \frac{4(x^2+y^2)}{w^4(z)}u_{n,m}(\xi,\eta) + 2C_{n,m}u_{n,m}(\xi,\eta) = 0 \tag{S.47}$$

$$\mathrm{i}k\frac{\mathrm{d}\phi'(z)}{\mathrm{d}z} + \frac{\mathrm{i}k}{R(z)}\phi'(z) = C_{n,m}\phi'(z) \tag{S.48}$$

where the constant $C_{n,m}$ is the eigenvalue of Eq. (S.47).

The mathematical analogy between number states and Hermite-Gaussian beams

To clearly unveil the analogy between number states and HG beams, we derive the dimensionless transverse spatial eigenmode equation and dimensionless time-independent harmonic equation.

The Eq. (S.47) describes the transverse pattern of HG beams, analogous to the time-independent harmonic equation Eq. (S.11). After making variable substitution $x \to \xi$ and $y \to \eta$, the dimensionless form of Eq. (S.47) could be written as:

$$\nabla_{\xi,\eta}^2 u_{n,m}(\xi,\eta) - (\xi^2+\eta^2)u_{n,m}(\xi,\eta) + 2C'u_{n,m}(\xi,\eta) = 0 \tag{S.49}$$

where $C' = C_{n,m}/(\sqrt{2}/w(z))$, $\nabla_{\xi,\eta}^2 = \frac{\partial^2}{\partial\xi^2} + \frac{\partial^2}{\partial\eta^2}$. We note that $u_{n,m}(\xi,\eta)$ as $|u_{n,m}\rangle$, $\tilde{p}_j = -\mathrm{i}\frac{\partial}{\partial j}$, $\tilde{q}_j = j$ in Cartesian coordinate, where $j = \xi,\eta$, then the Eq. (S.49) could be expressed as:

$$\tilde{\mathcal{H}}^{\mathrm{SL}}|u_{n,m}\rangle = C'|u_{n,m}\rangle \tag{S.50}$$

where $\tilde{\mathcal{H}}^{\mathrm{SL}} = \sum_{j=\xi,\eta}\left(\tilde{p}_j^2 + \tilde{q}_j^2\right)/2$. The set of $|u_{n,m}\rangle$ forms a full orthonormal basis and any two-mode quantum state can be represented as a superposition of $|u_{n,m}\rangle$ states.

The parameters (x', y') would be used to derive the dimensionless time-independent harmonic equation, where $x' = \sqrt{\omega/\hbar}x$ and $y' = \sqrt{\omega/\hbar}y$. Based on $\frac{\mathrm{d}^2}{\mathrm{d}x^2} = (\omega/\hbar)\frac{\mathrm{d}^2}{\mathrm{d}x'^2}$ and $\frac{\mathrm{d}^2}{\mathrm{d}y^2} = (\omega/\hbar)\frac{\mathrm{d}^2}{\mathrm{d}y'^2}$, the dimensionless form of Eq. (S.11) could be rewritten as:

$$\nabla_{x',y'}^2\psi(x',y') - (x'^2+y'^2)\psi(x',y') + 2E'\psi(x',y') = 0 \tag{S.51}$$

where $\psi(x',y')$ is the number state $|n,m\rangle$ in coordinate representation, $\nabla^2_{x',y'} = \frac{\partial^2}{\partial x'^2} + \frac{\partial^2}{\partial y'^2}$, $E' = E/(\hbar\omega)$. We note that $\hat{p'}_j = -\mathrm{i}\frac{\partial}{\partial j}$, $\hat{q'}_j = j$ in Cartesian coordinate, where $j = x', y'$, then the Eq. (S.51) could be expressed as:

$$\tilde{\mathcal{H}}' |n,m\rangle = E' |n,m\rangle \tag{S.52}$$

where $\hat{\mathcal{H}}' = \sum_{j=x',y'} \left(\hat{p'}_j^2 + \hat{q'}_j^2\right)/2$ is the dimensionless Hamiltonian operator. The set of $|n,m\rangle$ forms a full orthonormal basis and any two-mode quantum state can be represented as a superposition of $|n,m\rangle$ states.

We note that the dimensionless transverse spatial eigenmode equation Eq. (S.49) and the dimensionless time-independent Harmonic equation Eq. (S.51) share the same mathematical form and both of them constitute infinite-dimensional orthonormal spaces. Consequently, direct mapping can be established, that is $|n,m\rangle \leftrightarrow |u_{n,m}\rangle$. This correspondence strongly implies the existence of a classical analogy of a diverse family of quantum states where $|u_{n,m}\rangle$ modes play a role of $|n,m\rangle$ states. A summary table clarifies the analogies between quantum states and structured light, as shown in TABLE S1.

The Eq. (S.48) is the propagation evolution equation for paraxial structured light, playing the similar role as the time evolution equation Eq. (S.2) for quantum states. As for the time evolution operator of a quantified electromagnetic field $\hat{T} = \exp(-\mathrm{i}\hat{\mathcal{H}}t/\hbar)$, it satisfies $\mathrm{i}\hbar\frac{\mathrm{d}}{\mathrm{d}t}\hat{T} = E_{n,m}\hat{T}$. As for the propagation of structured light, we could define an analogous operator $\hat{Z}(z) = \mathrm{i}k\left[\frac{\mathrm{d}}{\mathrm{d}z} + \frac{1}{R(z)}\right]$, which satisfies $\hat{Z}(z)\phi'(z) = C_{n,m}\phi'(z)$. Although there are some differences, under a certain circumstance ($R(z) \to \infty$), these two equations are identical. Thus, the longitudinal coordinate z mimics the quantum variable t partly, which has been demonstrated in the main text.

STRUCTURED LIGHT ANALOGY OF SQUEEZED STATES

Structured light analogy of the squeezed vacuum state

Structured light of the single-mode squeezed vacuum state. Squeezed vacuum state (SVS) could be defined as applying the squeezed operator on vacuum state. There are single-mode and two-mode states. The single-mode SVS could be defined as [1]:

$$|\tau\rangle_0^{\text{single}} = \hat{S}_1(\tau)|0\rangle \tag{S.53}$$

where $|\tau\rangle_0^{\text{single}}$ represents the single-mode SVS, $|0\rangle$ represents the single-mode vacuum state, and $\hat{S}_1(\tau)$ is the single-mode squeezed operator defined as [1]:

$$\hat{S}_1(\tau) = \mathrm{e}^{\frac{1}{2}\left(\tau^*\hat{a}^2 - \tau\hat{a}^{\dagger 2}\right)} \tag{S.54}$$

where $\tau = |\tau| \arg\tau$ is a complex parameter. The expansion of Eq. (S.53) is [1]:

$$|\tau\rangle_0^{\text{single}} = \frac{1}{\sqrt{\cosh|\tau|}} \sum_{K=0}^{\infty} (-1)^K \frac{\sqrt{(2K)!}}{2^K K!} \mathrm{e}^{\mathrm{i}K\theta} \tanh^K|\tau| |2K\rangle \tag{S.55}$$

where $\theta = \arg\tau$. In order to construct the structured light analogy of single-mode SVS, we need to substitute the single-mode number state $|2K\rangle$ with the transverse pattern of single-mode HG beams $u_{2K}(\xi)$ firstly as:

$$\psi_0^{\text{single}}(\xi|\tau) = \frac{1}{\sqrt{\cosh|\tau|}} \sum_{K=0}^{\infty} (-1)^K \frac{\sqrt{(2K)!}}{2^K K!} \mathrm{e}^{\mathrm{i}K\theta} \tanh^K|\tau| u_{2K}(\xi) \tag{S.56}$$

Then adding the propagation factor of single-mode HG beams $\phi_1(z) = \left[\frac{2}{w^2(z)}\right]^{1/4} \exp\left[\mathrm{i}k\left(z + \frac{x^2}{2R(z)}\right) - \mathrm{i}\,(n+1/2)\tan^{-1}(\frac{z}{z_R})\right]$, the complete structured light analogy of single-mode SVS could be expressed in (x, z)-coordinate as:

$$\Psi_0^{\text{single}}(x,z|\tau) = \frac{1}{\sqrt{\cosh|\tau|}} \left[\frac{2}{\pi w^2(z)}\right]^{\frac{1}{4}} \mathrm{e}^{-\frac{x^2}{w^2(z)}} \mathrm{e}^{\mathrm{i}k\left[z + \frac{x^2}{2R(z)}\right]} \sum_{K=0}^{\infty} \frac{(-1)^K \mathrm{e}^{\mathrm{i}K\theta} \tanh^K|\tau|}{2^{2K}K!} H_{2K}\left[\frac{\sqrt{2}x}{w(z)}\right] \mathrm{e}^{-\mathrm{i}\left(2K+\frac{1}{2}\right)\tan^{-1}\left(\frac{z}{z_R}\right)} \tag{S.57}$$

Structured light analogy of the single-mode displaced squeezed vacuum state. Displaced squeezed vacuum state (DSVS) could be defined as applying the displaced and squeezed operators on the vacuum state. The single-mode DSVS could be defined as [1]:

$$|\alpha,\tau\rangle = \hat{D}(\alpha)\,\hat{S}(\tau)\,|0\rangle = \frac{1}{\sqrt{\cosh|\tau|}} \exp\left[-\frac{1}{2}|\alpha|^2 - \frac{1}{2}\alpha^{*2}\mathrm{e}^{\mathrm{i}\theta}\tanh|\tau|\right] \sum_{K=0}^{\infty} \frac{\left(\frac{1}{2}e^{\mathrm{i}\theta}\tanh|\tau|\right)^{K/2}}{\sqrt{K!}} H_K\left[\gamma\left(\mathrm{e}^{\mathrm{i}\theta}\sinh(2|\tau|)\right)^{-1/2}\right] |K\rangle \tag{S.58}$$

where $\gamma = \alpha\cosh|\tau| + \alpha^* e^{\mathrm{i}\theta}\sinh|\tau|$. In order to construct the structured light analogy of single-mode DSVS, we need to substitute the single-mode number state $|K\rangle$ with the transverse pattern of single-mode HG beams $u_K(\xi)$

TABLE S1: The analogy between quantum optics and classical paraxial light.

quantum optics		classical paraxial light	
Equation	$\left[\nabla^2_{x',y'} - (x'^2+y'^2) + 2E'\right]\psi(x',y') = 0$	Equation	$\left[\nabla^2_{\xi,\eta} - (\xi^2+\eta^2) + 2C'\right]u(\xi,\eta) = 0$
Harmonic operator	$\hat{\mathcal{H}} = -\frac{1}{2}\nabla^2_{x',y'} + \frac{1}{2}(x'^2+y'^2)$	Analogous operator	$\hat{\mathcal{H}}^{\mathrm{SL}} = -\frac{1}{2}\nabla^2_{\xi,\eta} + \frac{1}{2}(\xi^2+\eta^2)$
Wave function	$\psi_{n,m}(x',y')$	Wave function	$u^{\mathrm{HG}}_{n,m}(\xi,\eta)$
Eigenvalue	E'	Eigenvalue	C'
Coordinate	$(x',y') = \sqrt{\frac{\omega}{\hbar}}(x,y)$	Coordinate	$(\xi,\eta) = \frac{\sqrt{2}}{w(z)}(x,y)$
Parameter	$\sqrt{\frac{\omega}{\hbar}}$	Parameter	$\frac{\sqrt{2}}{w(z)}$
Probability	$\lvert\psi_{n,m}(x',y')\rvert^2$	Intensity	$\lvert u^{\mathrm{HG}}_{n,m}(\xi,\eta)\rvert^2$

firstly as:

$$\psi_0^{\mathrm{single}}(\xi|\alpha,\tau) = \frac{1}{\sqrt{\cosh|\tau|}} \mathrm{e}^{-\frac{1}{2}|\alpha|^2 - \frac{1}{2}\alpha^{*2}\mathrm{e}^{\mathrm{i}\theta}\tanh|\tau|} \sum_{K=0}^{\infty} \frac{\left(\frac{1}{2}e^{\mathrm{i}\theta}\tanh|\tau|\right)^{K/2}}{\sqrt{K!}} H_K\left[\gamma\left(\mathrm{e}^{\mathrm{i}\theta}\sinh(2|\tau|)\right)^{-1/2}\right] u_K(\xi) \tag{S.59}$$

Then adding the propagation factor of single-mode HG beams, the complete structured light analogy of single-mode DSVS could be expressed in (x, z)-coordinate as:

$$\Psi_0^{\mathrm{single}}(x,z|\alpha,\tau) = \frac{1}{\sqrt{\cosh|\tau|}} \mathrm{e}^{-\frac{1}{2}|\alpha|^2 - \frac{1}{2}\alpha^{*2}\mathrm{e}^{\mathrm{i}\theta}\tanh|\tau|} \left[\frac{2}{\pi w^2(z)}\right]^{\frac{1}{4}} \mathrm{e}^{-\frac{x^2}{w^2(z)}} \mathrm{e}^{\mathrm{i}k\left[z+\frac{x^2}{2R(z)}\right]} \sum_{K=0}^{\infty} \frac{\left(e^{\mathrm{i}\theta}\tanh|\tau|\right)^{K/2}}{2^K K!} H_K\left[\gamma\left(\mathrm{e}^{\mathrm{i}\theta}\sinh(2|\tau|)\right)^{-1/2}\right] H_K\left[\frac{\sqrt{2}x}{w(z)}\right] \mathrm{e}^{-\mathrm{i}\left(K+\frac{1}{2}\right)\tan^{-1}\left(\frac{z}{z_R}\right)} \tag{S.60}$$

Structured light analogy of the two-mode squeezed vacuum state. The two-mode SVS could be defined as [1]:

$$|\tau\rangle_0 = \hat{S}(\tau)|0,0\rangle \tag{S.61}$$

where $|\tau\rangle_0$ represents two-mode SVS, $|0,0\rangle$ represents two-mode vacuum state, $\hat{S}(\tau)$ is the two-mode squeezed operator defined as [1]:

$$\hat{S}(\tau) = \mathrm{e}^{\left(\tau^*\hat{a}\hat{b} - \tau\hat{a}^\dagger\hat{b}^\dagger\right)} \tag{S.62}$$

The expansion of Eq. (S.61) is [1]:

$$|\tau\rangle_0 = \frac{1}{\cosh r}\sum_{K=0}^{\infty}(-1)^K \mathrm{e}^{\mathrm{i}K\theta}\tanh^K|\tau|\,|K,K\rangle \tag{S.63}$$

The transverse pattern of its structured light analogy is:

$$\psi_0(\xi,\eta|\tau) = \frac{1}{\cosh|\tau|}\sum_{K=0}^{\infty}(-1)^K \mathrm{e}^{\mathrm{i}K\theta}\tanh^K|\tau| u_{K,K}(\xi,\eta) \tag{S.64}$$

And then adding the propagation factor $\phi(z)$, the complete structured light analogy of two-mode SVS could be expressed in (x, y, z)-coordinate as:

$$\Psi_0(x,y,z|\tau) = \frac{1}{\sqrt{\pi}w(z)\cosh|\tau|} \mathrm{e}^{-\frac{x^2+y^2}{w^2(z)}} \mathrm{e}^{\mathrm{i}k\left[z+\frac{x^2+y^2}{2R(z)}\right]} \sum_{K=0}^{\infty} \frac{(-1)^K \mathrm{e}^{\mathrm{i}K\theta}\tanh^K|\tau|}{\sqrt{2^{2K-1}(K!)^2}} H_K\left[\frac{\sqrt{2}x}{w(z)}\right] H_K\left[\frac{\sqrt{2}y}{w(z)}\right] \mathrm{e}^{-\mathrm{i}(2K+1)\tan^{-1}\left(\frac{z}{z_R}\right)} \tag{S.65}$$

Structured light analogy of two-mode displaced squeezed vacuum state. The two-mode DSVS could be defined as [1]:

$$\begin{aligned} |\alpha,\tau\rangle &= \hat{D}(\alpha)\hat{S}(\tau)|0,0\rangle \\ &= \frac{1}{\cosh|\tau|}\sum_{K=0}^{\infty}(-1)^K\exp(\mathrm{i}K\theta)(\tanh|\tau|)^K\left(1+|\alpha|^2\right)^{-K} \sum_{m=0}^{K}\frac{\sqrt{(K+m)!}(-\alpha^*)^m}{m!\sqrt{(K-m)!}}\sum_{m_1=0}^{K+m}\frac{\alpha^{m_1}}{m_1!}\frac{\sqrt{(K-m+m_1)!}\sqrt{(K+m)!}}{\sqrt{(K-m)!}\sqrt{(K+m-m_1)!}} \\ &\quad |K-m+m_1, K+m-m_1\rangle \end{aligned}, \tag{S.66}$$

Its corresponding transverse pattern of structured light is:

$$\psi_0(\xi,\eta|\alpha,\tau) = \frac{1}{\cosh|\tau|}\sum_{K=0}^{\infty}(-1)^K\exp(\mathrm{i}K\theta)(\tanh|\tau|)^K\left(1+|\alpha|^2\right)^{-K} \sum_{m=0}^{K}\frac{\sqrt{(K+m)!}(-\alpha^*)^m}{m!\sqrt{(K-m)!}}\sum_{m_1=0}^{K+m}\frac{\alpha^{m_1}}{m_1!}\frac{\sqrt{(K-m+m_1)!}\sqrt{(K+m)!}}{\sqrt{(K-m)!}\sqrt{(K+m-m_1)!}} u_{K-m+m_1,K+m-m_1}(\xi,\eta) \tag{S.67}$$

And then adding the propagation factor $\phi(z)$, the complete structured light analogy of two-mode DSVS could

be expressed in $(x,\, y,\, z)$-coordinate as:

$$\Psi_0(x,y,z|\alpha,\tau) = \frac{1}{\cosh|\tau|}\sum_{K=0}^{\infty}(-1)^K\exp\left(\mathrm{i}K\theta\right)\left(\tanh|\tau|\right)^K\left(1+|\alpha|^2\right)^{-K} \sum_{m=0}^{K}\frac{\sqrt{(K+m)!}(-\alpha^*)^m}{m!\sqrt{(K-m)!}}\frac{1}{\sqrt{\pi 2^{2K-1}}w(z)}\mathrm{e}^{-\frac{x^2+y^2}{w^2(z)}}\mathrm{e}^{\mathrm{i}k\left[z+\frac{x^2+y^2}{2R(z)}\right]} \sum_{m_1=0}^{K+m}\frac{\alpha^{m_1}}{m_1!}\frac{\sqrt{(K+m)!}}{\sqrt{(K-m)!(K+m-m_1)!}}H_{K-m+m_1}\left[\frac{\sqrt{2}x}{w(z)}\right] H_{K+m-m_1}\left[\frac{\sqrt{2}y}{w(z)}\right]\mathrm{e}^{-\mathrm{i}(2K+1)\tan^{-1}\left(\frac{z}{z_R}\right)} \tag{S.68}$$

Structured light analogy of squeezed number state

Structured light analogy of single-mode squeezed number state. Single-mode squeezed number state (SNS) was defined as [4]:

$$|\tau\rangle_N^{\text{single}} = \hat{S}_1(\tau)|N\rangle \tag{S.69}$$

where $|\tau\rangle_N^{\text{single}}$ represents single-mode SNS, $|N\rangle$ represents single-mode number state. The expansion of Eq. (S.69) is [4]:

$$|\tau\rangle_N^{\text{single}} = \left(\frac{1}{\cosh|\tau|}\right)^{N+\frac{1}{2}}\sqrt{N!}\sum_{j=0}^{[N/2]}\frac{\left[\tanh\left(|\tau|\right)\mathrm{e}^{-\mathrm{i}\theta}/2\right]^j\cosh^{2j}|\tau|}{(N-2j)!j!} \sum_{K=0}^{\infty}\frac{\left[-\tanh\left(|\tau|\right)\mathrm{e}^{\mathrm{i}\theta}/2\right]^K\sqrt{(N-2j+2K)!}}{K!}|N-2j+2K\rangle \tag{S.70}$$

Substitute the number state $|N-2j+2K\rangle$ with $u_{N-2j+2K}(\xi)$:

$$\psi_N^{\text{single}}(\xi|\tau) = \left(\frac{1}{\cosh|\tau|}\right)^{N+\frac{1}{2}}\sqrt{N!}\sum_{j=0}^{[N/2]}\frac{\left[\tanh\left(|\tau|\right)\mathrm{e}^{-\mathrm{i}\theta}/2\right]^j\cosh^{2j}|\tau|}{(N-2j)!j!} \sum_{K=0}^{\infty}\frac{\left[-\tanh\left(|\tau|\right)\mathrm{e}^{\mathrm{i}\theta}/2\right]^K\sqrt{(N-2j+2K)!}}{K!}u_{N-2j+2K}(\xi) \tag{S.71}$$

Considering the propagation factor of single-mode HG beams, the complete structured light analogy of single-mode SNS could be expressed in $(x,\, z)$-coordinate as:

$$\Psi_N^{\text{single}}(x,z|\tau) = \left(\frac{1}{\cosh|\tau|}\right)^{N+\frac{1}{2}}\left[\frac{2}{\pi w^2\left(z\right)}\right]^{\frac{1}{4}}\sqrt{N!}\mathrm{e}^{-\frac{x^2}{w^2(z)}} \times\mathrm{e}^{\mathrm{i}k\left[z+\frac{x^2}{2R(z)}\right]}\sum_{j=0}^{[N/2]}\frac{\left[\tanh\left(|\tau|\right)\mathrm{e}^{-\mathrm{i}\theta}/2\right]^j\cosh^{2j}|\tau|}{(N-2j)!j!} \sum_{K=0}^{\infty}\frac{\left[-\tanh\left(|\tau|\right)\mathrm{e}^{\mathrm{i}\theta}/2\right]^K}{K!\sqrt{2^{N-2j+2K}}}H_{N-2j+2K}\left[\frac{\sqrt{2}}{w\left(z\right)}x\right] \mathrm{e}^{-\mathrm{i}\left(N-2j+2K+\frac{1}{2}\right)\tan^{-1}\left(\frac{z}{z_R}\right)} \tag{S.72}$$

Structured light analogy of single-mode displaced squeezed number state. The single-mode displaced squeezed number state (DSNS) could be defined as [4]:

$$|\alpha,\tau\rangle_N^{\text{single}} = \hat{D}\left(\alpha\right)\hat{S}\left(\tau\right)|N\rangle = \left(\frac{1}{\cosh|\tau|}\right)^{N+1/2}\sqrt{N!}\exp\left(-\frac{|\alpha|^2}{2}\right) \sum_{j=0}^{[N/2]}\frac{[\tanh|\tau|\exp(-\mathrm{i}\theta)/2]^j(\cosh|\tau|)^{2j}}{(N-2j)!j!} \sum_{K=0}^{\infty}\frac{[-\tanh|\tau|\exp(\mathrm{i}\theta)/2]^K\sqrt{(N-2j+2K)!}}{K!} \sum_{n=0}^{N-2j+2K}\frac{(-\alpha^*)^n}{n!}\frac{\sqrt{(N-2j+2K)!}}{\sqrt{(N-2j+2K-n)!}} \sum_{m=0}^{\infty}\frac{\alpha^m}{m!}\frac{\sqrt{(N-2j+2K-n+m)!}}{\sqrt{(N-2j+2K-n)!}}|N-2j+2K-n+m\rangle \tag{S.73}$$

Substitute the number state $|N-2j+2K-n+m\rangle$ with $u_{N-2j+2K-n+m}(\xi)$:

$$\psi_N^{\text{single}}(\xi|\alpha,\tau) = \left(\frac{1}{\cosh|\tau|}\right)^{N+1/2}\sqrt{N!}\exp\left(-\frac{|\alpha|^2}{2}\right) \sum_{j=0}^{[N/2]}\frac{[\tanh|\tau|\exp(-\mathrm{i}\theta)/2]^j(\cosh|\tau|)^{2j}}{(N-2j)!j!} \sum_{K=0}^{\infty}\frac{[-\tanh|\tau|\exp(\mathrm{i}\theta)/2]^K\sqrt{(N-2j+2K)!}}{K!} \sum_{n=0}^{N-2j+2K}\frac{(-\alpha^*)^n}{n!}\frac{\sqrt{(N-2j+2K)!}}{\sqrt{(N-2j+2K-n)!}} \sum_{m=0}^{\infty}\frac{\alpha^m}{m!}\frac{\sqrt{(N-2j+2K-n+m)!}}{\sqrt{(N-2j+2K-n)!}}u_{N-2j+2K-n+m}(\xi) \tag{S.74}$$

Considering the propagation factor of single-mode HG beams, the complete structured light analogy of single-

mode DSNS could be expressed in (x, z)-coordinate as:

$$\Psi_N^{\text{single}}(x,z|\alpha,\tau)=\left(\frac{1}{\cosh|\tau|}\right)^{N+1/2}\sqrt{N!}\mathrm{e}^{-\frac{|\alpha|^2}{2}}\left[\frac{2}{\pi w^2(z)}\right]^{\frac{1}{4}}$$
$$\mathrm{e}^{-\frac{x^2}{w^2(z)}}\sum_{j=0}^{[N/2]}\frac{[\tanh|\tau|\exp(-\mathrm{i}\theta)/2]^j(\cosh|\tau|)^{2j}}{(N-2j)!j!}$$
$$\sum_{K=0}^{\infty}\frac{[-\tanh|\tau|\exp(\mathrm{i}\theta)/2]^K\sqrt{(N-2j+2K)!}}{K!}$$
$$\sum_{n=0}^{N-2j+2K}\frac{(-\alpha^*)^n}{n!}\frac{\sqrt{(N-2j+2K)!}}{\sqrt{(N-2j+2K-n)!}}$$
$$\sum_{m=0}^{\infty}\frac{\alpha^m}{m!\sqrt{2^{N-2j+2K-n+m}(N-2j+2K-n)!}}H_{N-2j+2K-n+m}\left[\frac{\sqrt{2}x}{w(z)}\right]$$
$$\mathrm{e}^{\mathrm{i}k\left[z+\frac{x^2}{2R(z)}\right]}\mathrm{e}^{-\mathrm{i}(N-2j+2K-n+m)\tan^{-1}\left(\frac{z}{z_R}\right)} \tag{S.75}$$

Structured light analogy of two-mode squeezed number state. The two-mode SNS could be defined as [4]:

$$|\tau\rangle_N=\hat{S}(\tau)|0,N\rangle \tag{S.76}$$

where $|\tau\rangle_N$ represents the two-mode SNS, $|0,N\rangle$ represents two-mode number state with N photons in the second mode and none in the first mode. The expansion of Eq. (S.76) is:

$$|\tau\rangle_N=$$
$$\frac{1}{\cosh|\tau|}\sum_{K=0}^{\infty}(-1)^K\mathrm{e}^{\mathrm{i}K\theta}\tanh^K(|\tau|)\sqrt{\frac{(N+K)!}{N!K!}}\,|K,N+K\rangle \tag{S.77}$$

Substitute the number state $|K,N+K\rangle$ with $u_{K,N+K}(\xi,\eta)$:

$$\psi_N(\xi,\eta|\tau)=$$
$$\frac{1}{\cosh|\tau|}\sum_{K=0}^{\infty}(-1)^K\mathrm{e}^{\mathrm{i}K\theta}\tanh^K(|\tau|)\sqrt{\frac{(N+K)!}{N!K!}}u_{K,N+K}(\xi,\eta) \tag{S.78}$$

Considering the propagation factor of two-mode HG beams, the complete structured light analogy of two-mode SNS could be expressed in (x, y, z)-coordinate as:

$$\Psi_N(x,y,z|\tau)=\frac{1}{\sqrt{\pi}w(z)\cosh|\tau|}\mathrm{e}^{-\frac{x^2+y^2}{w^2(z)}}\mathrm{e}^{\mathrm{i}k\left[z+\frac{x^2+y^2}{2R(z)}\right]}$$
$$\sum_{K=0}^{\infty}\frac{(-1)^K\mathrm{e}^{\mathrm{i}K\theta}\tanh^K(|\tau|)}{\sqrt{2^{N+2K-1}(N+K)!K!}}\sqrt{\frac{(N+K)!}{N!K!}}$$
$$H_K\left[\frac{\sqrt{2}x}{w(z)}\right]H_{N+K}\left[\frac{\sqrt{2}y}{w(z)}\right]\mathrm{e}^{-\mathrm{i}(2K+1)\tan^{-1}\left(\frac{z}{z_R}\right)} \tag{S.79}$$

Structured light analogy of two-mode displaced squeezed number state. The two-mode DSNS could be defined as [4]:

$$|\alpha,\tau\rangle_N=\hat{D}(\alpha)\hat{S}(\tau)|0,N\rangle$$
$$=\frac{1}{\cosh|\tau|}\sum_{K=0}^{\infty}(-1)^K\exp(\mathrm{i}K\theta)(\tanh|\tau|)^K\sqrt{\frac{(N+K)!}{N!K!}}$$
$$\left(1+|\alpha|^2\right)^{-\frac{N}{2}-K}\sum_{n=0}^{K}\frac{(-\alpha^*)^n\sqrt{K!}\sqrt{(N+K+n)!}}{n!\sqrt{(K-n)!}\sqrt{(N+K)!}}$$
$$\sum_{m=0}^{N+K+n}\frac{\alpha^m\sqrt{(K-n+m)!}\sqrt{(N+K+n)!}}{m!\sqrt{(K-n)!}\sqrt{(N+K+n-m)!}}$$
$$|K-n+m,N+K+n-m\rangle \tag{S.80}$$

To obtain the transverse pattern of the corresponding structured light, substitute the number state $|K-n+m,N+K+n-m\rangle$ with $u_{K-n+m,N+K+n-m}(\xi,\eta)$ as:

$$\psi_N(\xi,\eta|\alpha,\tau)=\frac{1}{\cosh|\tau|}\sum_{K=0}^{\infty}(-1)^K\exp(\mathrm{i}K\theta)(\tanh|\tau|)^K$$
$$\sqrt{\frac{(N+K)!}{N!K!}}\left(1+|\alpha|^2\right)^{-\frac{N}{2}-K}\sum_{n=0}^{K}\frac{(-\alpha^*)^n\sqrt{K!}\sqrt{(N+K+n)!}}{n!\sqrt{(K-n)!}\sqrt{(N+K)!}}$$
$$\sum_{m=0}^{N+K+n}\frac{\alpha^m\sqrt{(K-n+m)!}\sqrt{(N+K+n)!}}{m!\sqrt{(K-n)!}\sqrt{(N+K+n-m)!}}$$
$$u_{K-n+m,N+K+n-m}(\xi,\eta) \tag{S.81}$$

Considering the propagation factor of two-mode HG beams, the complete structured light analogy of two-mode DSNS could be expressed in (x, y, z)-coordinate as:

$$\Psi_N(x,y,z|\alpha,\tau)=\frac{1}{\sqrt{\pi}w(z)\cosh|\tau|}\mathrm{e}^{-\frac{x^2+y^2}{w^2(z)}}\mathrm{e}^{\mathrm{i}k\left[z+\frac{x^2+y^2}{2R(z)}\right]}$$
$$\sum_{K=0}^{\infty}(-1)^K\exp(\mathrm{i}K\theta)(\tanh|\tau|)^K$$
$$\sqrt{\frac{(N+K)!}{N!K!}}\left(1+|\alpha|^2\right)^{-\frac{N}{2}-K}\sum_{n=0}^{K}\frac{(-\alpha^*)^n\sqrt{K!}\sqrt{(N+K+n)!}}{n!\sqrt{(K-n)!}\sqrt{(N+K)!}}$$
$$\sum_{m=0}^{N+K+n}\frac{\alpha^m\sqrt{(N+K+n)!}}{m!\sqrt{2^{N+2K-1}(K-n)!(N+K+n-m)!}}$$
$$H_{K-n+m}\left[\frac{\sqrt{2}x}{w(z)}\right]H_{N+K+n-m}\left[\frac{\sqrt{2}y}{w(z)}\right]\mathrm{e}^{-\mathrm{i}(N+2K+1)\tan^{-1}\left(\frac{z}{z_R}\right)} \tag{S.82}$$

The wavefunction of quantum two-mode states

We exploit the wavefunction $\psi(q_1,q_2)$ in the dimensionless position basis (q_1,q_2) (the observables of the position operators of the jth mode $\hat{q}_j$, $j=1,2$) to characterize the quantum two-mode states, where the wavefunction $\psi(p_1,p_2)$ in the dimensionless momentum basis (p_1,p_2) (the observables of the momentum operators of the jth mode) is the Fourier transform of $\psi(q_1,q_2)$ [5]. The vacuum state and SVS and their structured light analogies have been discussed in the main text. Here, we exhibit the number state, SNS, DSVS and DSNS and their structured light analogies in Fig. S1.

The wavefunction of two-mode number state $|n, m\rangle$ in the dimensionless position basis is:

$$\psi_{n,m}(q_1, q_2) = \frac{1}{\sqrt{\pi 2^{n+m} n! m!}} H_n(q_1) H_m(q_2) \exp\left(-\frac{q_1^2 + q_2^2}{2}\right), \tag{S.83}$$

which would be reduced to the wavefunction of two-mode vacuum state for $n = m = 0$. The probability distribution of $|\psi_{n,m}(q_1, q_2)|^2$ with $(n, m) = (0, 6)$, corresonding to number state $|0, N\rangle$ with $N = 6$, is plotted in the left subfigure of Fig. S1 **a1**, while its right subfigure shows the probability density distribution with varying θ on the direction of the observable $\hat{q}_2$. θ defines the orientation in the coordinate (q_1, q_2). The noise of number state is marked with black dashed lines, noticing that it is not the standard quantum limit (SQL). SQL only refers to the noise of vacuum state. In the structured light regime, we map the number state $|n, m\rangle$ to HG mode $|u_{n,m}\rangle$. The transverse profiles of HG mode at various planes are shown in Fig. S1 **b1**, where its beam waist (marked with white dashed lines) is regarded as the counterpart for the noise of number state. The longitudinal evolution of HG mode is illustrated in Fig. S1 **c1**. Due to the diffraction of light, though, the distribution becomes broader for structured light as it propagates, we plot the intensity of structured light in the reduced coordinates $(x/w(z), y/w(z))$ where $w(z)$ is the beam waist dependent on z.

The wavefunction of two-mode squeezed states could be squeezed by a factor $R/\sqrt{2}$ and enlarged by a factor $\sqrt{2}R$ in the quadrature directions [5]. Therefore, the wavefunction of two-mode SNS in the dimensionless position basis could be represented as:

$$\psi_{sqn}(q_1, q_2) = \frac{1}{\sqrt{\pi 2^{n+m} n! m!}} H_n\left(\frac{q_1}{\sqrt{2}R}\right) H_m\left(\frac{Rq_2}{/\sqrt{2}}\right) \times \exp\left(-\frac{q_1^2}{4R^2} - \frac{R^2 q_2^2}{4}\right) \tag{S.84}$$

where applying the rotation matrix in the **Methods** on it could manipulate the squeezing direction. The probability density distribution of $|\psi_{sqn}(q_1, q_2)|^2$ with $(n, m) = (0, 6)$ and $R = exp(0.5)$, corresponding to SNS $\hat{S}(\tau)|0, N\rangle$ with $N = 6$ and $\tau = 0.5$, is shown in the left subfigure of Fig. S1 **a2**, where its noise is marked with blue dashed lines. The noise is squeezed in the direction of the observable $\hat{q}_2$, less than the noise of number state but not surpassing SQL. The noise of SNS is also enlarged in the quadrature direction of the observable $\hat{q}_1$. Besides, the noise in the direction of the observable $\hat{q}_2$ oscillates with θ varying at a period of π (the right subfigure of Fig. S1 **a2**). The transverse profiles of the structured light analogy of SNS at various planes are shown in Fig. S1 **b2** where its beam waist is marked with blue dashed lines. Its spot size is smaller than the HG mode on the direction marked with blue arrows, while larger in the quadrature direction, akin to squeezing noise of SNS. The longitudinal evolution of the structured light analogy of SNS is illustrated in Fig. S1 **c2**, where the squeezing direction varies in propagation direction z. This behavior qualitatively reproduces that of the SNS, where the squeezing direction varies (oscillates) with θ.

The displaced operator $\hat{D}(\alpha)$ would cause an overall displacement of the wavefunction distribution in the phase space as $q_j \rightarrow q_j + \mathrm{Re}(\sqrt{2}\alpha)$ and $q_j \rightarrow p_j + \mathrm{Im}(\sqrt{2}\alpha)$, where Re(.) and Im(.) represent the real and imaginary parts of the parameter, respectively [5]. Figs. S1 **a3** and **a4** exhibit the probability density distributions of DSVS and DSNS with $\alpha = 0.5$. The intensity distributions for their structured light analogies at various planes are shown in Fig. S1 **b3** and **b4**, where its beam waist is marked with blue dashed lines. It should be noticed that these generalized cases (number state, SNS, DSVS and DSNS) are just interesting theoretical expansion, since their abilities of surpassing SQL in quantum domain and surpassing SSL in structured light counterpart are weaker than that of SVS. But the structured light analogies of these generalized squeezed states enrich the structured light family and the quantum-classical correspondence cases, which may have potential applications in communication since they have more degrees of freedom such as the quantum number N and the displaced parameter α.

Q function for quantum single-mode states

The mathematical expression of Q function. Q function was introduced in quantum optics as the expectation value of the density operator in a coherent state, which is defined as [6]:

$$Q(\beta) = \frac{1}{\pi} |\langle \beta | \tau \rangle|^2 \tag{S.85}$$

where $|\tau\rangle$ represents the characterized quantum state and $\langle \beta|$ represents the left vector of coherent state, β and τ are complex parameters. The coherent state $|\beta\rangle$ is defined as [6]:

$$|\beta\rangle = \mathrm{e}^{-\frac{1}{2}|\beta|^2} \sum_{K=0}^{\infty} \frac{\beta^K}{\sqrt{K!}} |K\rangle \tag{S.86}$$

Then we would derive the Q function of the above quantum states. For vacuum state:

$$Q_0(\beta) = \frac{1}{\pi} |\langle \beta | 0 \rangle|^2 = \frac{1}{\pi} \mathrm{e}^{-|\beta|^2} \tag{S.87}$$

For SVS:

$$Q_0^s(\beta) = \frac{1}{\pi} |\langle \beta | \tau \rangle_0|^2 = \frac{1}{\pi \cosh |\tau|} \mathrm{e}^{-|\beta|^2 - \frac{1}{2}[\beta^{*2} \exp(\mathrm{i}\theta) + \beta^2 \exp(-\mathrm{i}\theta)] \tanh |\tau|} \tag{S.88}$$

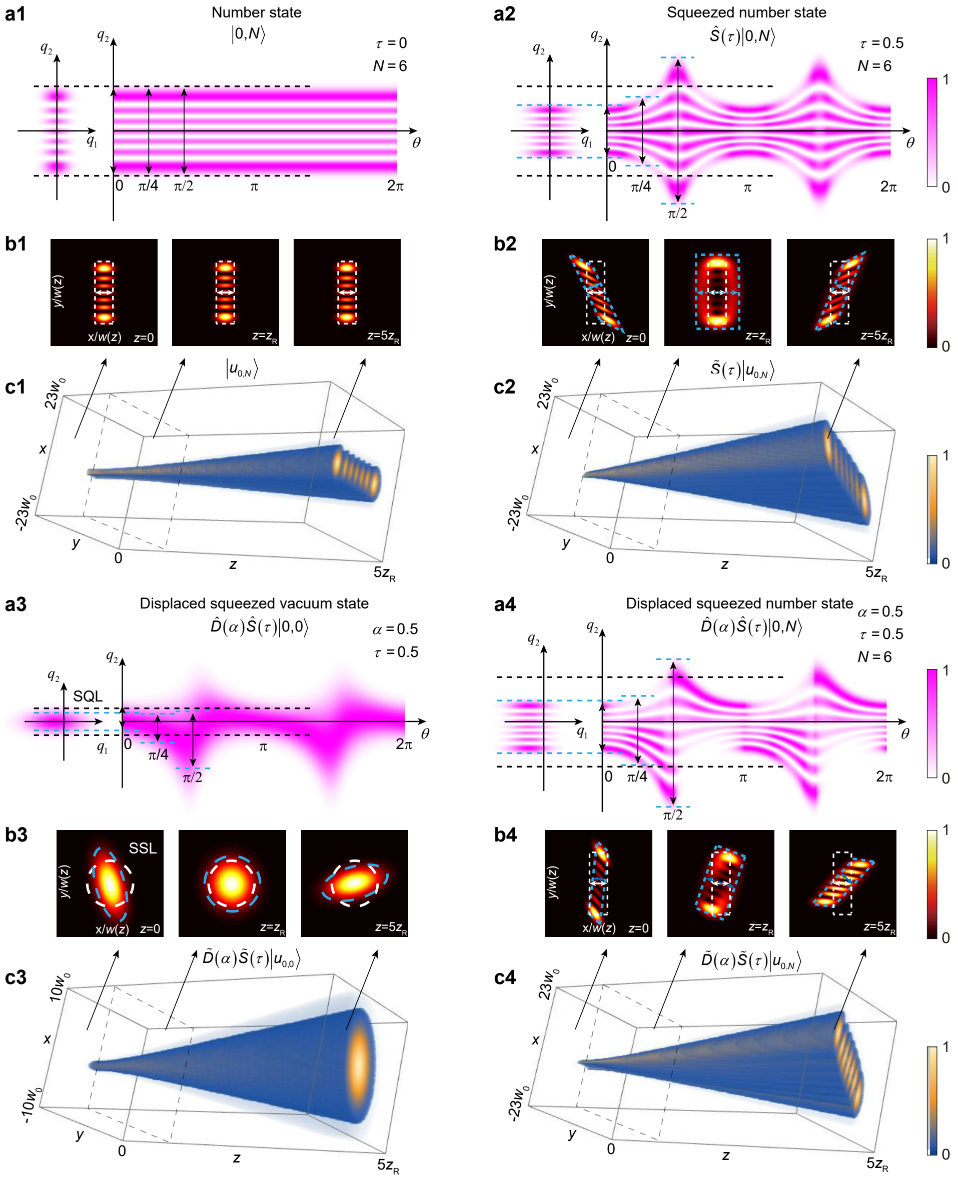

FIG. S1: **Quantum squeezed states and their structured light analogies.** **a** The quantum probability density distributions for **a1** number state, **a2** squeezed number state, **a3** displaced squeezed vacuum state and **a4** displaced squeezed number state, where the black dashed lines mark the noise of the vacuum state (in **a3**) and number state (in **a1**, **a2** and **a4**), the blue dashed lines mark the noise of squeezed states, θ ranges from 0 to 2π, q_1 and q_2 are two quadratu filed quadratures (canonical coordinates). **b** The transverse modes of the corresponding structured light analogies (simulation) at different z, where $(x/w(z), y/w(z))$ ranges from -5 to 5 for **a1**, **a2** and **a4**, and from -2 to 2 for **a3**. The white dashed lines mark the waist of Gaussian modes (in **a3**) and HG modes (in **a1**, **a2** and **a4**). The blue dashed lines mark the waist of the structured light analogy of suqeezed states. **c** The propagation evolution of the corresponding structured light analogies, where z ranges from 0 to $5z_\mathrm{R}$, (x, y) ranges from $-23w_0$ to $23w_0$ for **a1**, **a2** and **a4**, and from $-10w_0$ to $10w_0$ for **a3**, w_0 is the beam waist at $z = 0$, z_R is the Rayleigh range. SQL: standard quantum limit; SSL: standard spatial limit. Colormap: darkness to brightness means 0 to 1.

For DSVS:

$$Q_0^{ds}(\beta) = \frac{1}{\pi}\left|\langle\beta|\alpha,\tau\rangle_0\right|^2$$

$$\langle\beta|\alpha,\tau\rangle_0 =$$

$$\frac{1}{\sqrt{\cosh|\tau|}}\exp\left[-\frac{1}{2}|\alpha|^2 - \frac{1}{2}|\beta|^2 - \frac{1}{2}\alpha^{*2}e^{\mathrm{i}\theta}\tanh|\tau|\right]$$

$$\sum_{n=0}^{\infty}\frac{\left(\frac{1}{2}e^{\mathrm{i}\theta}\beta^{*2}\tanh|\tau|\right)^{n/2}}{n!}H_n\left[\gamma\left(e^{\mathrm{i}\theta}\sinh\left(2|\tau|\right)\right)^{-1/2}\right] \tag{S.89}$$

For number state:

$$Q_N(\beta) = \frac{1}{\pi}\left|\langle\beta|N\rangle\right|^2 = \frac{1}{\pi}\mathrm{e}^{-|\beta|^2}\frac{|\beta|^{2N}}{N!} \tag{S.90}$$

For SNS:

$$Q_N^s(\beta)=\frac{1}{\pi}\left|\langle\beta|\tau\rangle_N\right|^2,$$

$$\langle\beta|\tau\rangle_N=\mathrm{e}^{-\frac{|\beta|^2}{2}}\left(\frac{1}{\cosh|\tau|}\right)^{N+1/2}\mathrm{e}^{\frac{-\tanh(|\tau|)\exp(\mathrm{i}\theta)\beta^{*2}}{2}}\sqrt{N!}$$
$$\sum_{K=0}^{[N/2]}\frac{\left[\tanh\left(|\tau|\right)\exp\left(-\mathrm{i}\theta\right)/2\right]^K\left(\cosh|\tau|\right)^{2K}\left(\beta^*\right)^{N-2K}}{(N-2K)!K!} \tag{S.91}$$

For DSNS:

$$Q_N^{ds}(\beta)=\frac{1}{\pi}\left|\langle\beta|\alpha,\tau\rangle_N\right|^2$$

$$\langle\beta|\alpha,\tau\rangle_N=\left(\frac{1}{\cosh|\tau|}\right)^{n+1/2}\sqrt{N!}\exp\left(-\frac{|\alpha|^2+|\beta|^2}{2}\right)$$
$$\sum_{j=0}^{[N/2]}\frac{\left[\tanh\left(|\tau|\right)\exp\left(-\mathrm{i}\theta\right)/2\right]^j\left(\cosh|\tau|\right)^{2j}}{(N-2j)!j!}$$
$$\sum_{k=0}^{\infty}\frac{\left[-\tanh\left(|\tau|\right)\exp\left(\mathrm{i}\theta\right)/2\right]^k\sqrt{(N-2j+2k)!}}{k!}$$
$$\sum_{n=0}^{N-2j+2k}\frac{(-\alpha^*)^n\sqrt{(N-2j+2k)!}}{n!\sqrt{(N-2j+2k-n)!}}$$
$$\sum_{m=0}^{\infty}\frac{\alpha^m\beta^{N-2j+2k-n+m}}{m!\sqrt{(N-2j+2k-n)!}} \tag{S.92}$$

Astigmatic Gaussian beams

The intensity distribution of astigmatic Gaussian beams could demonstrate a similar behavior as the probability distribution of two-mode squeezed states. The beam waists of a Gaussian beam focused by a single cylindrical lens would be anisotropic, which could be expressed as [7]:

$$w_x(z)=w_0\sqrt{1+\left(\frac{z-f}{f}\right)^2} \tag{S.93}$$

$$w_y(z)=w_0\sqrt{1+\left(\frac{z+f}{f}\right)^2} \tag{S.94}$$

where w_0 is the beam waist of Gaussian before going through the single cylindrical lens, f is the focal length of the cylindrical lens, (x,y) are the active and inactive axes of the cylindrical lens. Then, the amplitude distribution of the astigmatic Gaussian beams could be expressed as:

$$\psi_0(x,y,z)=\frac{1}{\sqrt{\pi}}\exp\left[-\frac{x^2}{w_x^2(z)}-\frac{y^2}{w_y^2(z)}\right] \tag{S.95}$$

which is similar to the wavefunction of two-mode squeezed states. The Eq. (10) in the main text would be reduced to $\Psi_{\mathrm{sq}}(q_1,q_2)=\frac{1}{\sqrt{\pi}}\exp\left[-q_1^2/(4R^{-2})-q_2^2(4R^2)\right]$ when $\theta_0=-\pi/4$, where $R=\exp(|\tau|)$. The parameter relation between the wavefunction and the astigmatic Gaussian beam could be obtained from $R^4=w_y^2(z)/w_x^2(z)$, which could be derived as:

$$|\tau|=\frac{1}{4}\ln\left[\frac{f^2+(z+f)^2}{f^2+(z-f)^2}\right] \tag{S.96}$$

which reveals that the propagation variable z and the focus length f tune the squeezing amplitude $|\tau|$. It should be noted that the value of the right part of Eq. (S.96) ranges from 0 to about 0.45, meaning that only the structured light analogy of squeezed states in the limited range of $|\tau|$ could be realized via the astigmatic Gaussian beams. Furthermore, the astigmatic lens cannot continually tune the parameters of the structured light analogy of squeezed states.

THE GENERATION AND DETECTION OF STRUCTURED LIGHT ANALOGY OF SQUEEZED STATES

The simulation and experimental results for the generation and detection of structured light analogy of squeezed states with various parameters τ and N are shown in this section, where N ranges from 0 to 6 with the step size of 2, $|\tau|$ ranges from 0 to 0.5 with the step size of 0.2, $\arg\tau$ ranges from 0 to π with the step size of $\pi/4$. Since the modal spectra are same for various $\arg\tau$, i.e. independent on $\arg\tau$, the case of $\arg\tau=0$ is taken as an example in the simulation and experiment.

The hologram of generating classical squeezed structured light. The classical squeezed structured light is generated by a hologram (mask) loaded on SLM1, as shown in Fig. S2.

The experimental results of structured light analogy of squeezed number state. We generate the structured light analogy of squeezed number state and detect its modal spectra in experiment, as an extended case of the squeezed vacuum state in the main text, as shown in Fig. S3. Subfigures **a** present the varying squeezing direction. Subfigure **b** shows the intensity profile of the structured light analogy of squeezed number state at the $\zeta-z$ plane, where the gray dashed lines mark the spot size of HG modes $|u_{0,N}\rangle$ and the blue dashed lines mark the spot size of the structured light analogy of squeezed number state $\tilde{S}(\tau)\,|u_{0,N}\rangle$. The spot size of $\tilde{S}(\tau)\,|u_{0,N}\rangle$ is smaller than that of $|u_{0,N}\rangle$, exhibiting the squeezing effect but not surpassing SSL. Subfigure **c** shows the detected modal spectra with *cor* $=0.965$ and $F=0.856$, which is larger than that in Fig. 3 in the main text, since the errors would be enlarged with increasing τ. The corresponding probability density distribution is illustrated in Fig. S3 **d**, where the gray dashed lines mark

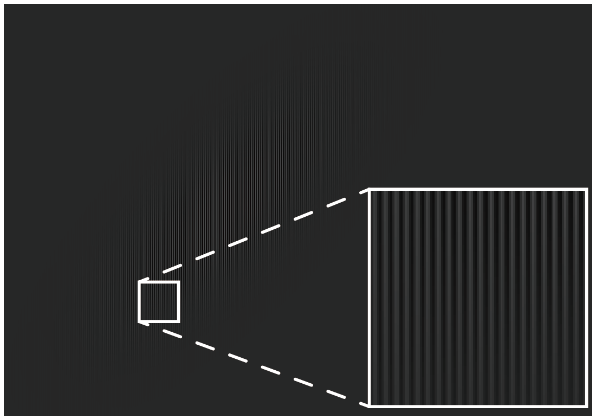

FIG. S2: **The hologram of generating classical squeezed structured light.** The left area marked with a white box is zoomed in the right area marked with a white box.

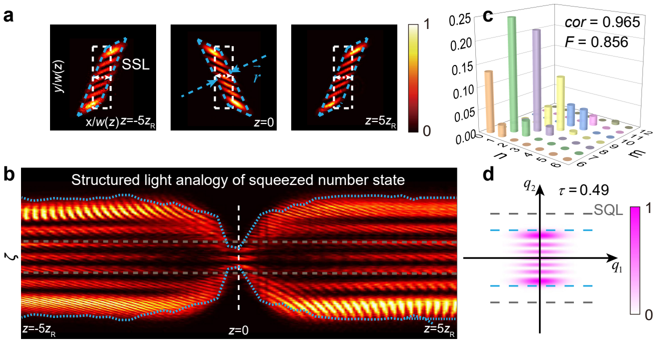

FIG. S3: **Experimental structured light analogy of squeezed number state.** **a** The transverse modes of the structured light analogy of squeezed number state with $z = -5z_{\mathrm{R}}$, 0, and $5z_{\mathrm{R}}$, respectively. The blue arrows mark the squeezing direction at $z = 0$. The blue dashed lines mark the beam waist of the structured light analogies. The white dashed lines mark the beam waist of HG beam $|u_{0,6}\rangle$. **b** The intensity distributions at $\zeta - z$ plane, where z ranges from $-5z_{\mathrm{R}}$ to $5z_{\mathrm{R}}$, ζ is the normalized radial position along the squeezing direction at $z = 0$. **c** The detected modal spectrum. **d** The corresponding probability density distributions, with $\tau = 0.49$. *cor*: the correlation coefficient between the simulations and experimental results, ranging from 0 to 1. F: the fidelity between the simulations and experimental results, ranging from 0 to 1. Colormap: darkness to brightness means 0 to 1.

the noise of number state and the blue dashed lines mark the noise of the corresponding squeezed number state, presenting less noise but not surpassing SQL. That is why the squeezed number state including the other two generalized squeezed states are just discussed as interesting theoretical expansion.

The transverse pattern and modal spectrum of structured light analogy of squeezed states. Fig. S4 shows the simulation (top section) and experimental results (bottom section) for the structured light analogy of single-mode SNS, where the left figure is the transverse pattern of structured light at $z = 0$ plane, x ranges from $-8w_0$ to $8w_0$, and the right figure is the corresponding modal spectrum, respectively. Fig. S5 shows the simulation (top section) and experimental results (bottom section) for the structured light analogy of two-mode SNS, where the left figure is the transverse pattern of structured light at $z = 0$ plane, (x, y) ranges from $-6w_0$ to $6w_0$, and the right figure is the corresponding modal spectrum, respectively. On the horizontal direction, the structured light analogy of SNS would reduce to the structured light analogy of SVS with N tending to 0. On the vertical direction, the structured light analogy of SNS would reduce to the structured light analogy of number state, with τ tending to 0. On the diagonal direction, the structured light analogy of SNS would reduce to the structured light analogy of vacuum state, with both N and τ tending to 0.

The transverse patterns of structured light analogy of squeezed states with various τ. Fig. S6 shows the simulation (left column) and experimental results (right column) for the transverse patterns of structured light analogy of single-mode squeezed states at $z = 0$ plane, where N ranges from 0 to 6, corresponding to the evolution from single-mode SVS to single-mode SNS. Fig. S7 shows the simulation (left column) and experimental results (right column) for the transverse patterns of structured light analogy of two-mode squeezed states at $z = 0$ plane, where N ranges from 0 to 6, corresponding to the evolution from two-mode SVS to two-mode SNS. Each subsection shows the transverse patterns of structured light with various τ.

The propagation of structured light analogy of squeezed states. Fig. S8 shows the simulation (left column) and experimental results (right column) for the propagation of structured light analogy of single-mode squeezed states, Fig. S9 shows the simulation (left column) and experimental results (right column) for the propagation of structured light analogy of two-mode squeezed states, where z ranges from 0 to $2z_{\mathrm{R}}$, N ranges from 0 to 6 corresponding to the evolution from single-mode SVS to single-mode SNS.

TIGHTLY FOCUSING RESULTS

Tightly focusing simulation. The simulation of tightly focused structured light analogy of SVS with various parameters τ are shown in Fig. S10, where τ ranges from 0 to 1 with the step size of 0.2. The left figures are the tightly focused transverse patterns at $z = 0$ plane, where $(x,\ y)$ ranges from -2λ to 2λ. The waist of tightly focused structured light analogy of SS would evolve from a circle (marked with gray dotted circles) to an ellipse (marked with blue dotted ellipses). The blue and green curves mark the squeezed and quadra-

ture directions, which reveals that the size of tightly focused spots would be decreased in the squeezed direction (marked with blue arrows) but enlarged in the quadrature direction (marked with green arrows). The right figures show the intensity distributions on the diagonal directions, where the intensity distributions are same at squeezed and quadrature directions in the case of $\tau = 0$, but decreased and enlarged with increasing τ, respectively, which reveals that the tightly focused structured light analogy of SVS could be applied in super-oscillatory at the squeezed direction by tuning the parameter τ.

Phase retrieval. We exploit the Gerchberg-Saxton algorithm for the phase retrieval of the tightly focused patterns, which is illustrated in Fig. S11. We recorded two intensity profiles of tightly focused patterns, noted as I_1 and I_2, in a distance of about 1 μm that $z_1 - z_2 = 1$ μm. The phase profiles in the $j-$th iteration are noted as ph_1 and ph_2, respectively. The initial ph_1 could be selected as simulation. The initial field ψ_1 at the z_1 plane is $\psi_1 = \sqrt{I_1}\exp(\mathrm{i}ph_1)$. Then we exploit the angular spectrum method to compute the field $\psi_2 = \mathrm{ifft}\left[H(f_x, f_y)\mathrm{fft}(\psi_1))\right]$ at the z_2 plane, where fft and ifft are the Fourier transform and inverse Fourier transform, $H(f_x, f_y) = \exp\left(\mathrm{i}2\pi d\sqrt{\frac{1}{\lambda^2} - f_x^2 - f_y^2}\right)$, (f_x, f_y) are the frequency domain coordinates, $d = |z_1 - z_2|$ is the distance between two planes, $ph_2 = \mathrm{angle}(\psi_2)$. And then we exploit the recorded intensity profile $\sqrt{I_2}$ to modify that $\psi_2 = \sqrt{I_2}\exp(\mathrm{i}ph_2)$. Then we exploit the reverse angular spectrum method to reconstruct the field in the z_1 plane that $\psi_1 = \mathrm{ifft}\left[H^{-1}(f_x, f_y)\mathrm{fft}(\psi_2))\right]$, where $H^{-1}(f_x, f_y) = \exp\left(-\mathrm{i}2\pi d\sqrt{\frac{1}{\lambda^2} - f_x^2 - f_y^2}\right)$, $ph_1 = \mathrm{angle}(\psi_1)$. Here we also need the recorded I_1 to modify that $\psi_1 = \sqrt{I_1}\exp(\mathrm{i}ph_1)$ and repeat above process until we get a stable result.

Tightly focusing experiment. The schematic diagram of tightly focusing experiment is shown in Fig. S12, where the beam shaping part in the white dotted box is to generate the structured light analogies of SVS in free space which is tightly focused by the other parts. The process of beam shaping part is same as the generation part in the experiment of quantum-classical correspondence. The generated structured light analogies of SVS in free space is coupled in the microscope objective with large numerical aperture (NA) (labeled "NA") via a normal lens (L5). The microscope objective would tightly focus the paraxial beams on the reflector. Then, the reflector would transform the tightly focused patterns into the camera through the microscope objective and the lens L6. The lens L6 and the microscope objective are assembled as a $4f$ system with $60\times$ magnification. The camera is used to record the patterns.

Acknowledgment

This work is supported by Beijing Natural Science Foundation (JQ23021); the National Research Foundation, Singapore and A∗STAR under the Quantum Engineering Programme (QEP-P1 and NRF2021-QEP2-01-P01). Y. Shen acknowledges the support from Nanyang Technological University Start Up Grant, Singapore Ministry of Education (MOE) AcRF Tier 1 grant (RG157/23), MoE AcRF Tier 1 Thematic grant (RT11/23). J. Y. Ou acknowledges the UK's Engineering and Physical Sciences Research Council (EP/T02643X/1) and the Royal Society (RG\R2\232531). The authors thank Dr. Cilong Zhang for the experimental assistance of the tightly focused structured light.

References

* Electronic address: qiangliu@tsinghua.edu.cn
† Electronic address: yijie.shen@ntu.edu.sg
‡ Electronic address: fuxing@tsinghua.edu.cn

[1] C. Gerry, P. Knight, and P. L. Knight, *Introductory quantum optics* (Cambridge university press, 2004).
[2] G. Nienhuis and L. Allen, Physical Review A **48**, 656 (1993).
[3] G. J. Gbur, *Mathematical Methods for Optical Physics and Engineering* (Cambridge University Press, 2011).
[4] M. M. Nieto, Physics Letters A **229**, 135 (1997), ISSN 0375-9601.
[5] A. I. Lvovsky, *Squeezed light* (2016), 1401.4118.
[6] W. P. Schleich, *Quantum States in Phase Space* (John Wiley & Sons, Ltd, 2001), chap. 4, pp. 99–151, ISBN 9783527602971.
[7] Y. F. Chen, C. C. Chang, C. Y. Lee, J. C. Tung, H. C. Liang, and K. F. Huang, Laser Physics **28**, 015002 (2017).

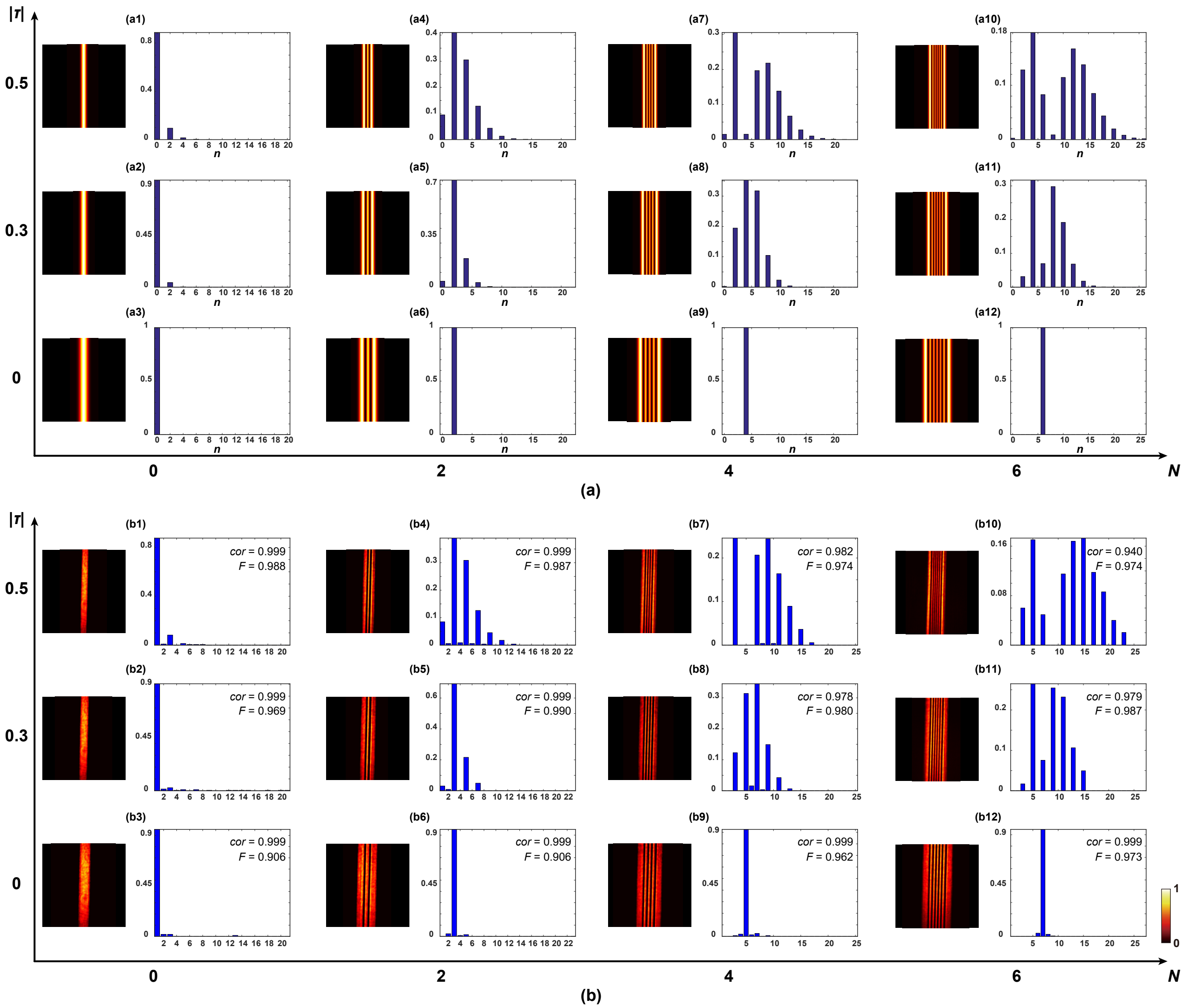

FIG. S4: **The modal spectra of structured light analogy of single-mode squeezed states**. The simulation (top row) and experiment (bottom row) for transverse patterns and modal spectra of structured light analogy of single-mode squeezed states at $z = 0$ plane. The horizontal axes for the patterns (left figures) are x that ranges from $-8w_0$ to $8w_0$. The horizontal axes for the modal spectra (right figures) are indices n that ranges from 0 to 10 for $N = 0$, from 0 to 12 for $N = 2$, from 0 to 14 for $N = 4$, from 0 to 16 for $N = 6$, respectively. *cor*: the correlation coefficients betweeen the simulation and the experimental results, ranging from 0 to 1. F: the fidelity between the simulations and experimental results, ranging from 0 to 1. Colormap: darkness to brightness means 0 to 1 for intensity.

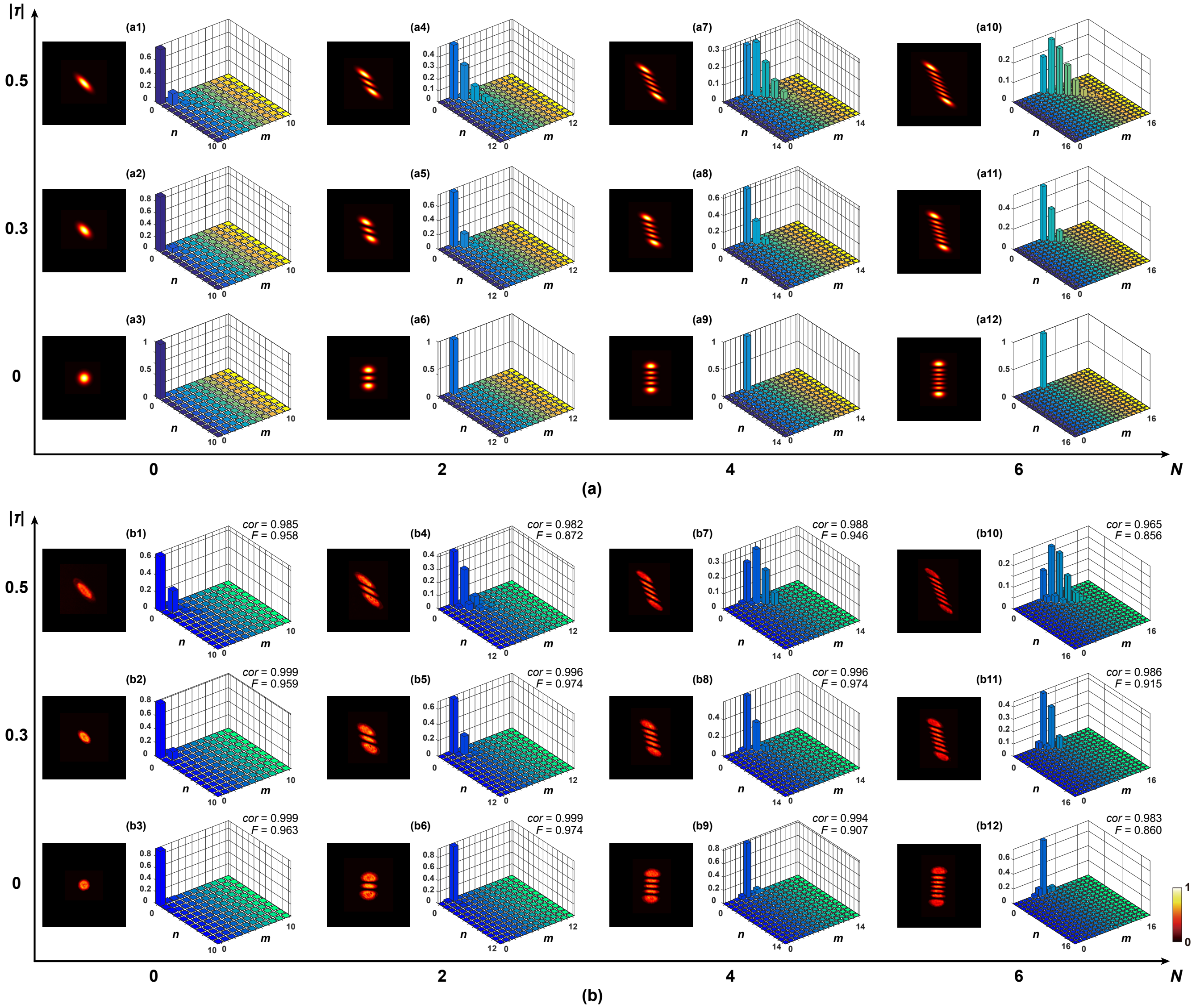

FIG. S5: **The modal spectra of structured light analogy of two-mode squeezed states**. The simulation (top row) and experiment (bottom row) for transverse patterns and modal spectra of structured light analogy of two-mode squeezed states at $z = 0$ plane. The horizontal and vertical axes for the patterns (left figures) are x and y-axis, respectively. (x, y) ranges from $-6w_0$ to $6w_0$. The horizontal and vertical axes for the modal spectra (right figures) are indices n and m, respectively. (n, m) ranges from 0 to 10 for $N = 0$, from 0 to 12 for $N = 2$, from 0 to 14 for $N = 4$, from 0 to 16 for $N = 6$, respectively. *cor*: the correlation coefficients betweeen the simulation and the experimental results, ranging from 0 to 1. F: the fidelity between the simulations and experimental results, ranging from 0 to 1. Colormap: darkness to brightness means 0 to 1 for intensity.

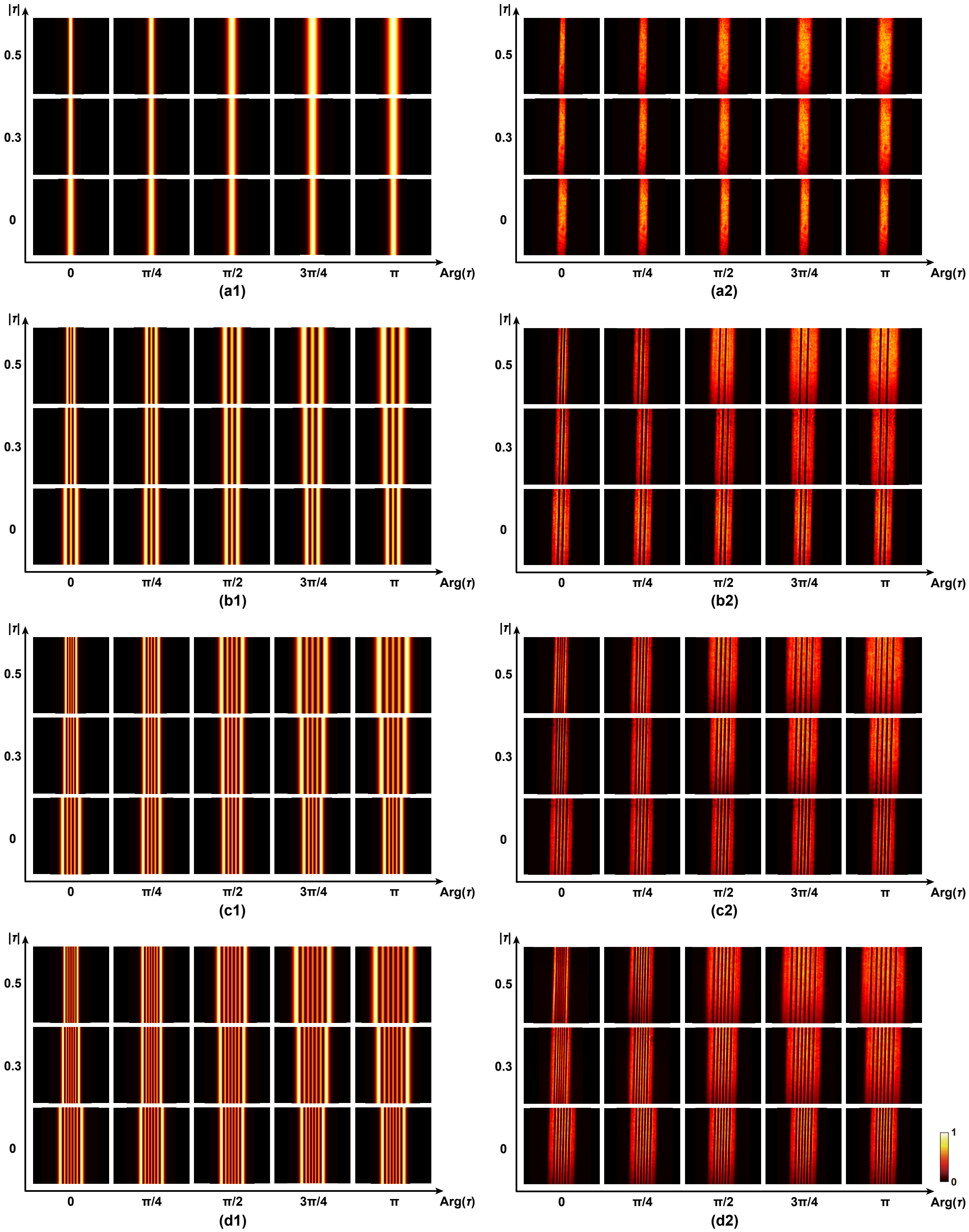

FIG. S6: **The transverse patterns of structured light analogy of single-mode squeezed states**. The simulation (left column) and the experimental results (right column) for transverse patterns of structured light analogy of single-mode squeezed states at $z = 0$ plane, with various N and τ. **a** The transverse patterns of structured light analogy of single-mode SVS. **b-d** The transverse patterns of structured light analogy of single-mode SNS with $N = 2$, 4, 6, respectively. The horizontal axe is x-axis and x ranges from $-8w_0$ to $8w_0$. Colormap: darkness to brightness means 0 to 1 for intensity.

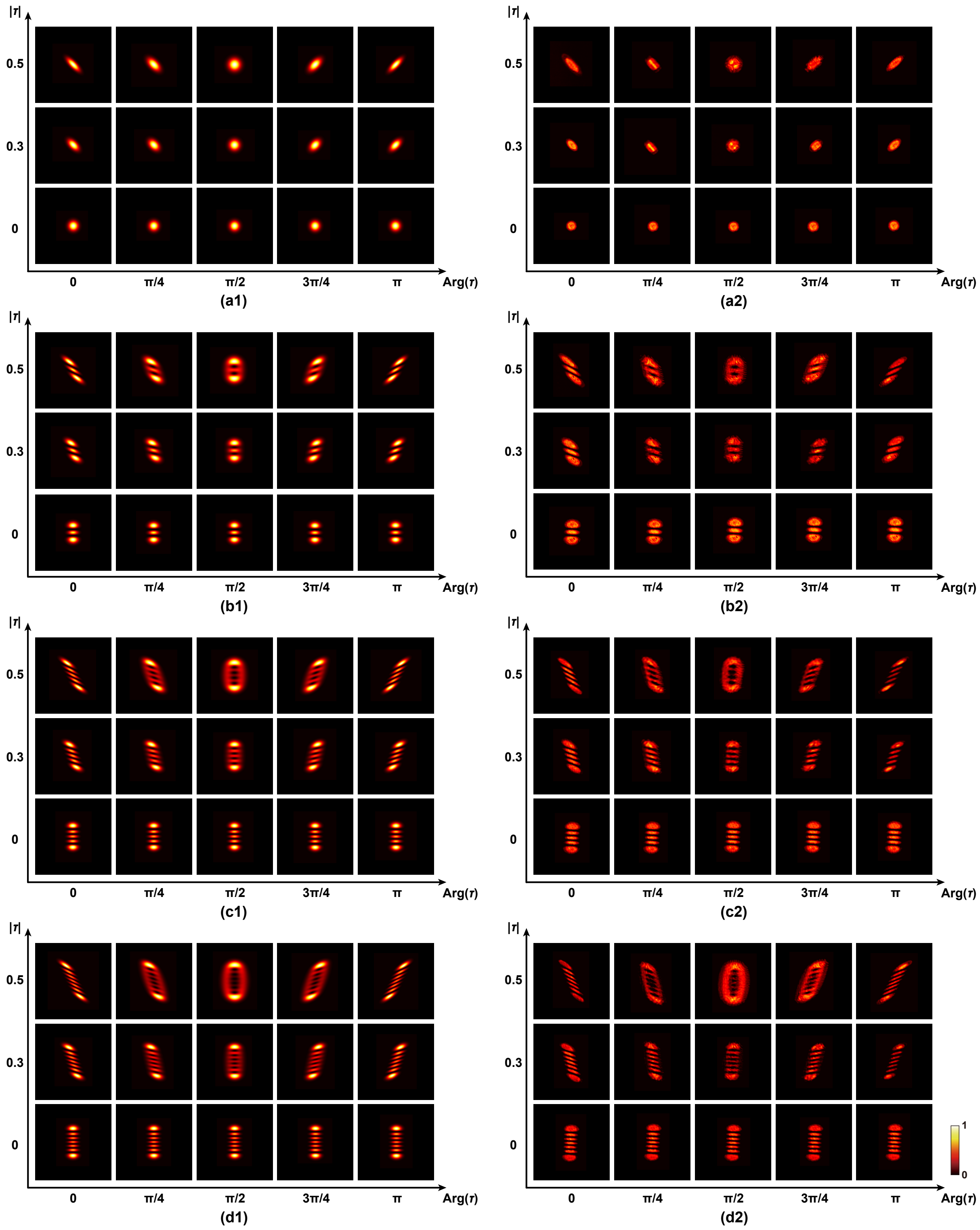

FIG. S7: **The transverse patterns of structured light analogy of two-mode squeezed states**. The simulation (left column) and the experimental results (right column) for transverse patterns of structured light analogy of two-mode squeezed states at $z = 0$ plane, with various N and τ. **a** The transverse patterns of structured light analogy of two-mode SVS. **b-d** The transverse patterns of structured light analogy of two-mode SNS with $N = 2$, 4, 6, respectively. The horizontal and vertical axes are x and y-axis, respectively. (x, y) ranges from $-6w_0$ to $6w_0$. Colormap: darkness to brightness means 0 to 1 for intensity.

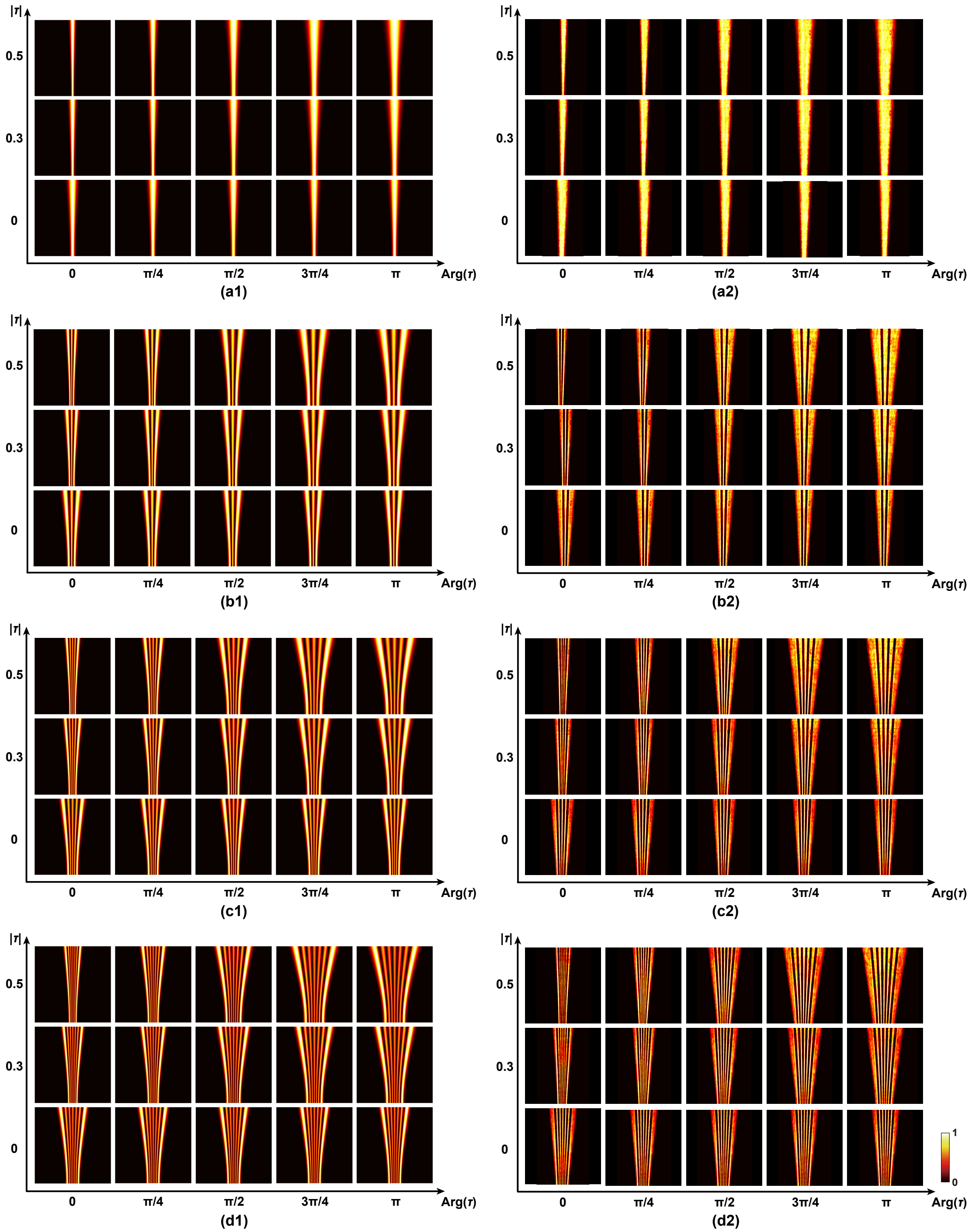

FIG. S8: **The propagation of structured light analogy of single-mode squeezed states**. The simulation (left column) and the experimental results (right column) for the propagation of structured light analogy of single-mode squeezed states. **a** The propagation of structured light analogy of single-mode SVS. **b-d** The propagation of structured light analogy of single-mode SNS with $N = 2, 4, 6$, repectively. The horizontal and vertical axes are x and z-axis, respectively. z ranges from 0 to $2z_R$, x ranges from $-7w(2z_R)$ to $7w(2z_R)$. Colormap: darkness to brightness means 0 to 1 for intensity.

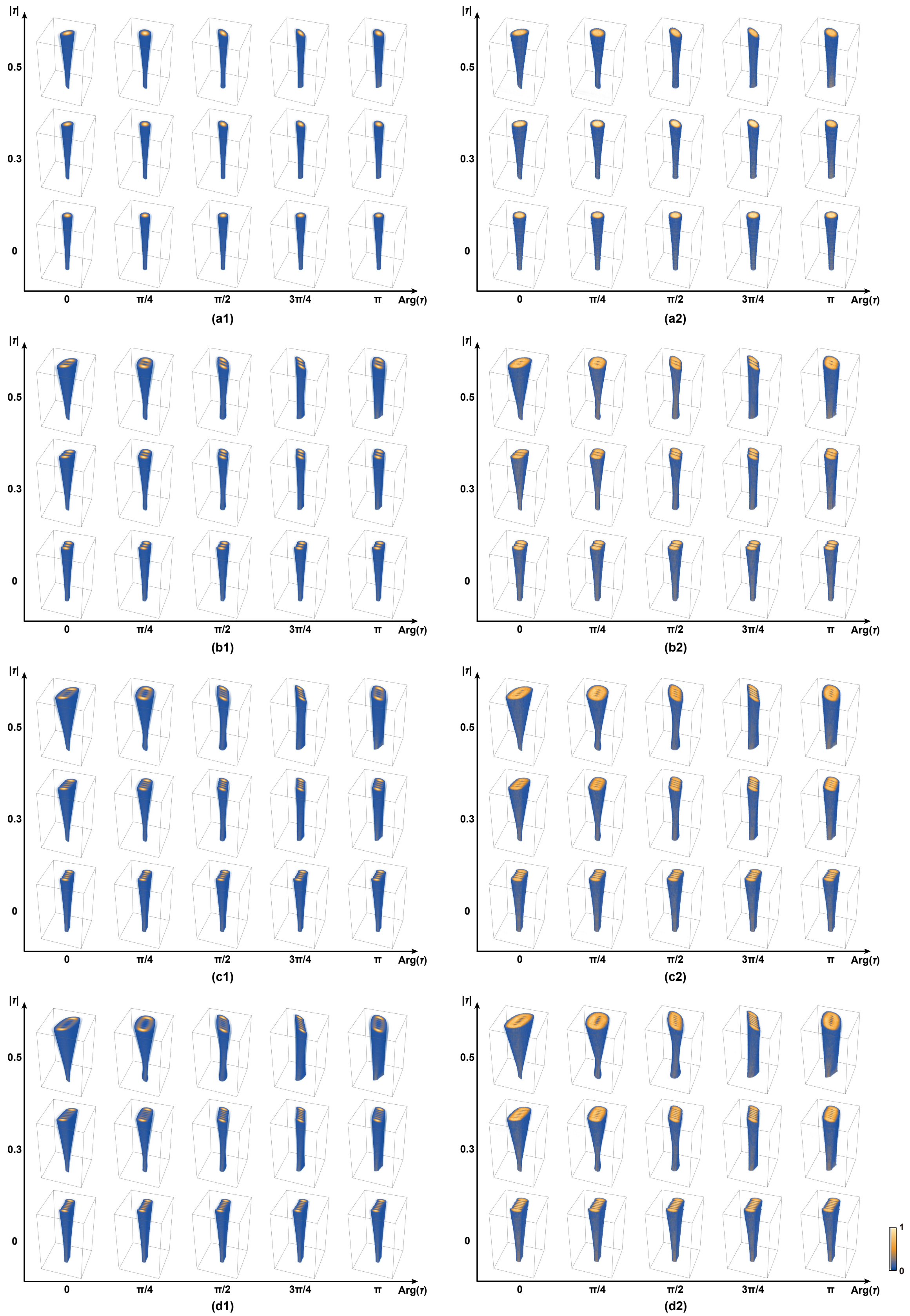

FIG. S9: **The propagation of structured light analogy of two-mode squeezed states**. The simulation (left column) and the experimental results (right column) for the propagation of structured light analogy of two-mode squeezed states. **a** The propagation of structured light analogy of two-mode SVS. **b-d** The propagation of structured light analogy of two-mode SNS with $N = 2$, 4, 6, respectively. z ranges from 0 to $2z_{\mathrm{R}}$, (x, y) ranges from $-6w(2z_{\mathrm{R}})$ to $6w(2z_{\mathrm{R}})$. Colormap: darkness to brightness means 0 to 1 for intensity.

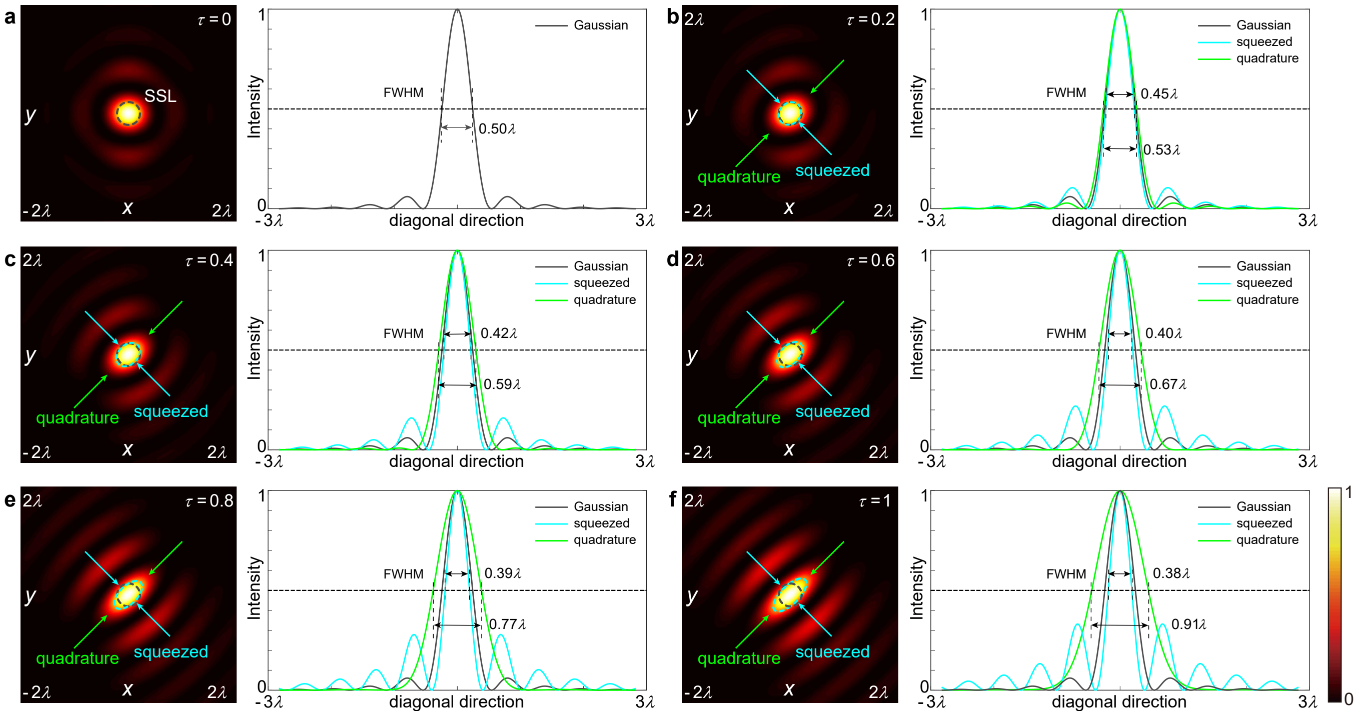

FIG. S10: **The simulation of tightly focused structured light analogy of SVS.** The left figures are the tightly focused transverse patterns at $z = 0$ plane, where (x, y) ranges from -2λ to 2λ, the blue and green curves mark the squeezed and quadrature directions, gray dotted circles mark the waist of tightly focused Gaussian beam, blue dotted ellipses mark the waist of tightly focused structured light analogy of squeezed states. The right figures are intensity distribution on the diagonal directions, where the black curve corresponds to the Gaussian beam, with the FWHM of the central spot $\lambda/2$, the blue and green curves corresponds to the intensity distributions at squeezed and quadrature directions, respectively. τ ranges from 0 to 1 with the step size of 0.2. Colormap: darkness to brightness means 0 to 1 for intensity.

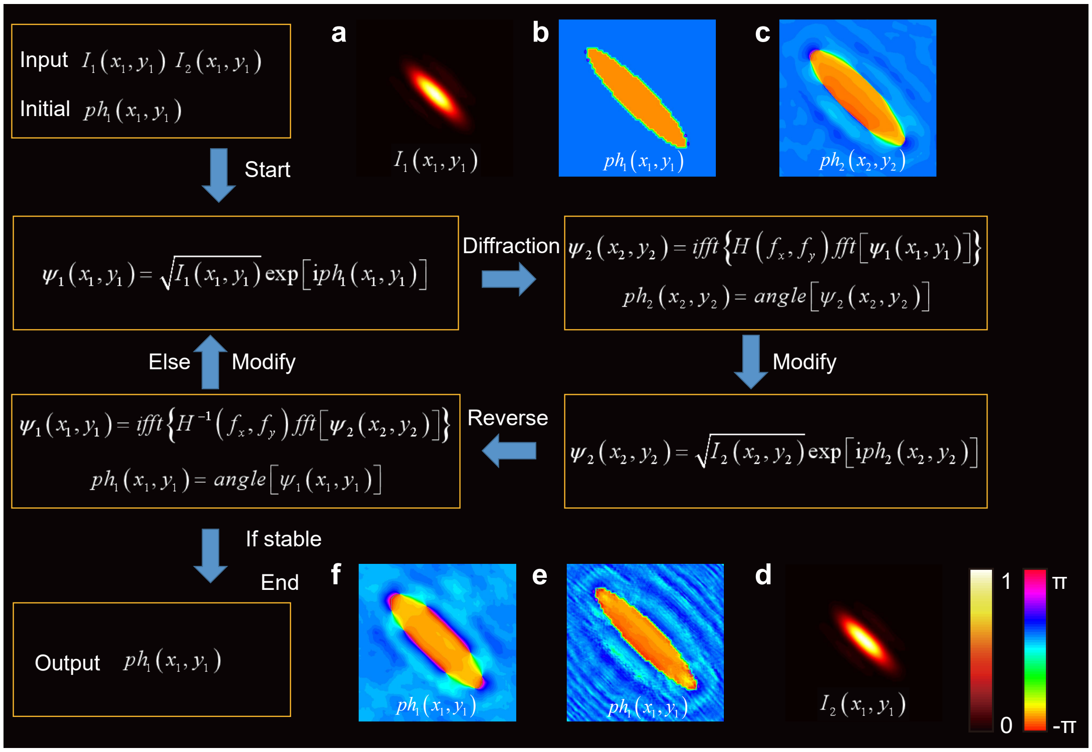

FIG. S11: **The phase retrieval process.** The top section with the black background maps the phase retrieval process, where the main idea is to iterate the phase distributions $ph_1(x_1, y_1)$ and $ph_2(x_2, y_2)$ in two planes based the experimentally recorded intensity profiles $I_1(x_1, y_1)$ and $I_2(x_2, y_2)$ in both planes. The intensity profiles **a** $I_1(x_1, y_1)$ and **d** $I_2(x_2, y_2)$ can be recorded by CCD in experiment. **b** The simulated phase at (x_1, y_1) plane as the initial phase $ph_1(x_1, y_1)$. **c** The computed phase $ph_2(x_2, y_1)$ at (x_2, y_2) plane in the first loop. **e** The computed phase $ph_1(x_1, y_1)$ at (x_1, y_1) plane in the first loop. **f** The computed phase $ph_1(x_1, y_1)$ at (x_1, y_1) plane in the 30th loop, which is the output result and used in the main text.

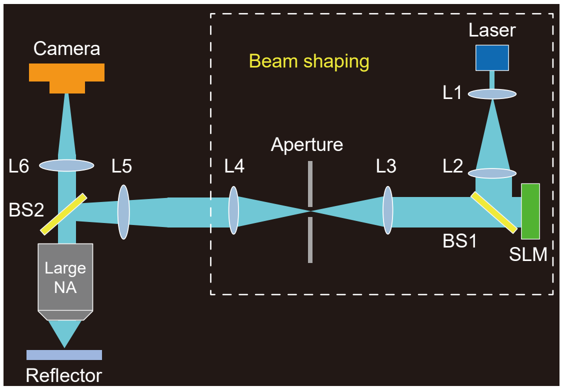

FIG. S12: **The schematic diagram of tightly focusing experiment.** SLM: liquid-crystal spatial light modulator; the gray box marked with "Large NA": tightly focused lens; BS: beam splitter; Camera: record the pattern; Reflector: reflect the tightly focused patterns.
